# Ultrasensitive Colorimetric Luminescence Thermometry by Progressive Phase Transition

**DOI:** 10.1002/advs.202305241

**Published:** 2023-12-11

**Authors:** Hao Suo, Dongxu Guo, Peihang Zhao, Xin Zhang, Yu Wang, Weilin Zheng, Panlai Li, Tao Yin, Li Guan, Zhijun Wang, Feng Wang

**Affiliations:** ^1^ National‐Local Joint Engineering Laboratory of New Energy Photoelectric Devices Hebei Key Laboratory of Optic‐electronic Information and Materials College of Physics Science & Technology Hebei University Baoding 071002 China; ^2^ Department of Materials Science and Engineering City University of Hong Kong Kowloon Hong Kong SAR 999077 China

**Keywords:** doping, lanthanide ions, luminescence material, phase transition, thermometry

## Abstract

Luminescent materials that display quick spectral responses to thermal stimuli have attracted pervasive attention in sensing technologies. Herein, a programmable luminescence color switching in lanthanide‐doped LiYO_2_ under thermal stimuli, based on deliberate control of the monoclinic (*β*) to tetragonal (*α*) phase transition in the crystal lattice, is reported. Specifically, a lanthanide‐doping (Ln^3+^) approach to fine‐tune the phase‐transition temperature in a wide range from 294 to 359 K is developed. Accordingly, an array of Ln^3+^‐doped LiYO_2_ crystals that exhibit progressive phase transition, and thus sequential color switching at gradually increasing temperatures, is constructed. The tunable optical response to thermal stimuli is harnessed for colorimetric temperature indication and quantitative detection, demonstrating superior sensitivity and temperature resolution (*S*_r_ = 26.1% K^−1^, *δT* = 0.008 K). The advances in controlling the phase‐transition behavior of luminescent materials also offer exciting opportunities for high‐performance personalized health monitoring.

## Graphical abstract

Ultrasensitive optical thermometry is realized over a broad temperature range through progressive phase transitions in LiYO_2_ crystals. Remarkably, a programmable luminescence color switching at prescribed temperatures is realized. Thus an ultra‐high thermal sensitivity and resolution that outperforms the existing luminescent thermometers is obtained. The superiority of this thermometer for intelligent wearable devices is substantiated.

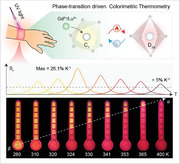

## 1 Introduction

Luminescent materials with thermo‐responsive spectroscopic properties have aroused significant interest in the thermometric field due to their applications in microelectronics, cell biology, and preclinical diagnostics.^[^ ^1^ ^]^ In particular, the self‐calibrated ratiometric concept, which leverages luminescence intensity ratio (LIR) as the temperature indicator, has become the mainstream technique in the current thermal sensing field due to its high resistance to environmental interference.^[^ ^2^ ^]^ To date, several LIR strategies have been developed to take advantage of dual emission bands from a wide collection of lanthanide and transition metal ions, in which thermal stimulation strongly affects the population of different electronic states in a single ion or a pair of ions.^[^ ^3^ ^]^ A well‐established example is the LIR sensor governed by the Boltzmann distribution law, which has been extensively studied with full knowledge of the physical model.^[^ ^4^ ^]^ However, these approaches typically suffer from insufficient thermal sensitivity (*S*_r_ < 5% K^−1^) and temperature resolution (*δT* > 0.1 K), hardly satisfying the ever‐increasing demands in advanced applications, particularly in complex environments. For example, the temperature variations associated with physiological processes are usually lower than 1 K, which requires a resolution of 0.1 K to accurately monitor the thermal status of the human body for daily healthcare, such as cardiovascular health, pulmonological diagnostics, cognitive state, and other syndromes.^[^ ^5^ ^]^

**Figure 1:**
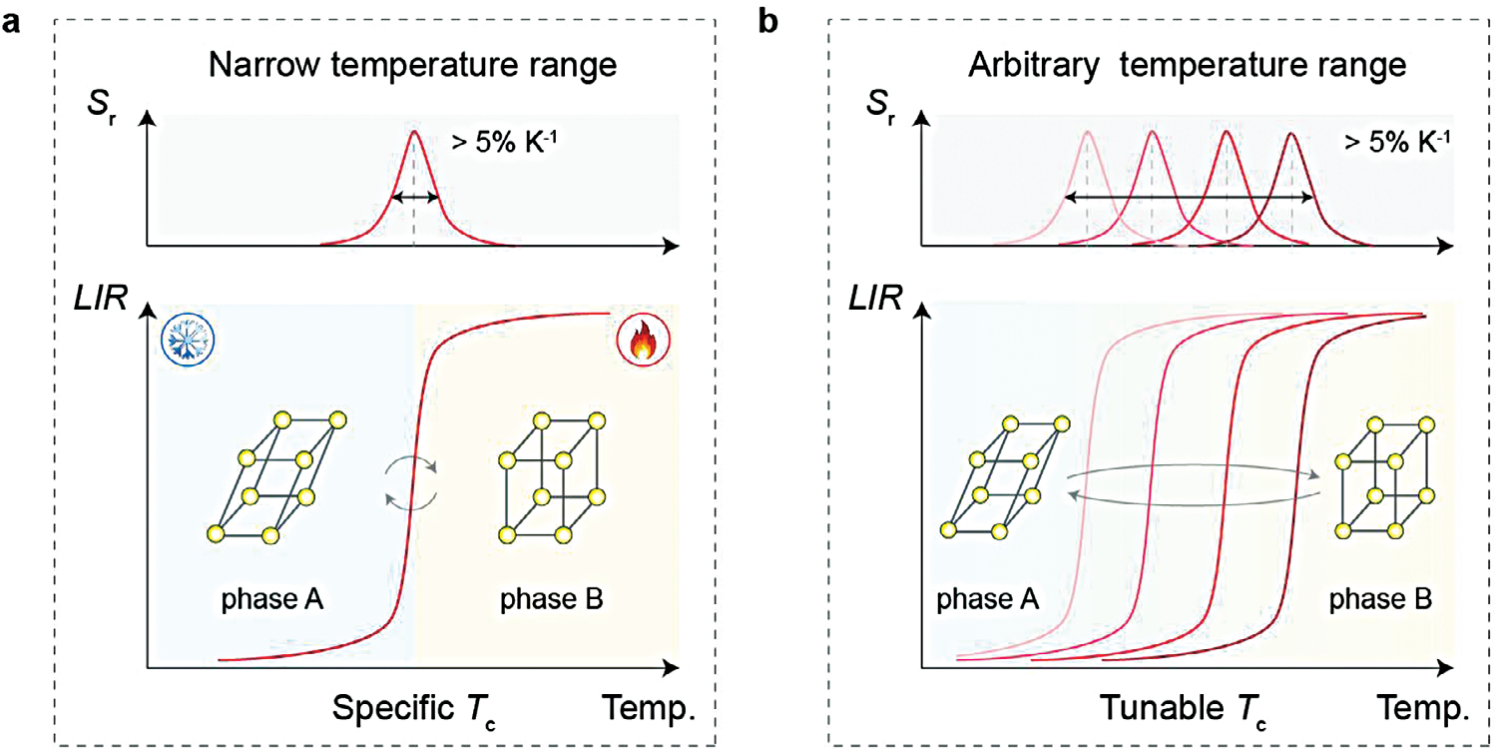
Schematic representation of phase‐transition‐driven thermometry. a) Reported LIR model that depends on the thermally induced reversible phase transition of luminescence materials. The distinct crystal phase and local structure trigger the ratiometric luminescence switching at a specific critical temperature, thereby enabling superior *S*_r_ (>5% K^−1^) within a narrow temperature range. b) Proposed LIR model driven by the customizable phase transition in LiYO_2_ crystals under thermal stimuli. The phase‐transition temperature can be precisely controlled by regulating the ionic radius at the rare‐earth site, thereby leading to superior *S*_r_ (>5% K^−1^) over extended temperature ranges.

The anomalous temperature dependence of crystal structure has enabled another principle of designing LIR thermometers for sensitive thermal detection. The 4*f* intra‐configurational transitions of lanthanide ions can be substantially tuned by the short‐range lattice structure (e.g., local symmetry, coordination, and interionic distance and angle), stemming from the co‐effects of electron repulsion, spin‐orbit coupling, and crystal field.^[^ ^6^ ^]^ Thermal stimulation may appreciably modify the crystallographic structure of materials by inducing structural distortions through thermal expansion.^[^ ^7^ ^]^ In some cases, intrinsic phase transformation occurs as the temperature varies across a specific point, resulting in a distinct change of luminescence property (e.g., intensity, wavelength shift, and lifetime) near the phase‐transition temperature.^[^ ^8^ ^]^ Although high thermal sensitivity has been obtained, thermometric approaches utilizing phase transition are largely unfulfilled due to the narrow working temperature range (**Figure** **1**a).^[^ ^9^ ^]^

In this study, we establish an efficient method for ultrasensitive thermal detection over a broad temperature range using phase‐transition‐engineered LiYO_2_:Pr^3+^ crystals (Figure 1b). We develop a lanthanide doping approach to fine‐tune the phase‐transition temperature of LiYO_2_:Pr^3+^ crystals in the 294–359 K range. Accordingly, we demonstrate sequential switching of Pr^3+^ luminescence in a series of LiYO_2_:Pr^3+^ crystals as a function of temperature, enabling high‐performance colorimetric thermal indication and quantification. We show that the ultrasensitive and adjustable spectral response to temperature can be harnessed for healthcare monitoring with a resolution lower than 0.1 K.

## 2 Results and Discussion

**Figure 2:**
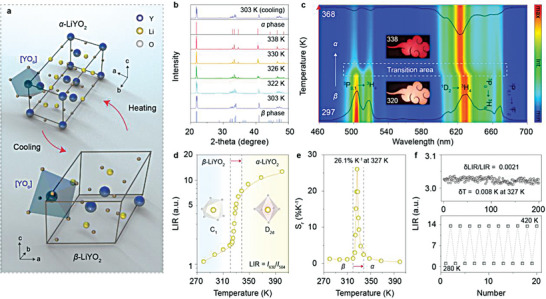
PL switching performance induced by phase transition. a) Schematic illustration of the thermally induced reversible phase transition between monoclinic (*β*) and tetragonal (*α*) phases of LiYO_2_ crystal. The [YO_6_] polyhedrons are highlighted in blue color. b) In situ XRD patterns of LiYO_2_:Pr^3+^ (0.25%) at different temperatures. c) Normalized PL spectral mapping as a function of temperature under 280 nm excitation along with the PL spectra at 297 and 368 K. Inset shows the luminescence images of the sample in an “auspicious clouds” pattern at the onset (320 K) and completion (338 K) temperatures of the PL switching process, respectively. d) The calculated LIR value at different temperatures. Inset shows the schematics of the [YO_6_] polyhedrons. Note that the LIR value was calculated by the ratio of integrated intensity in the 600–645 nm range (^1^D_2_ → ^3^H_4_) and 490–520 nm (^3^P_0,1_ → ^3^H_4_). e) The calculated *S*_r_ value as a function of temperature. f) Fluctuation of LIR values measured 200 times at 327 K, along with the reversibility test of the LIR values.

Our study employed lithium yttrium oxide (LiYO_2_) as the host material due to its high compatibility with lanthanide dopants. On top of that, LiYO_2_ crystal stabilizes in the monoclinic *β* phase (space group: *P*2_1_/*c*) with a point symmetry of *C*_1_ in the octahedral Y site at around room temperature (RT, 298 K), while transferring into the tetragonal *α* phase (space group: *I*4_1_/*amd*) with a point symmetry of *D*_2d_ in the octahedral Y site with the increase of temperature (**Figure** **2**a).^[^ ^10^ ^]^ Pr^3+^ ion was selected as the model dopant owing to its dual‐color emissions (^3^P_0,1_, ^1^D_2_ → ^3^H_4_) that are sensitive to the local crystal structure.^[^ ^11^ ^]^ In theory, Pr^3+^ ion (*r* = 0.99 Å) occupies the octahedral Y^3+^ site (*r* = 0.9 Å) due to the close ionic radii and same valence state, thereby subject to varying crystal fields in a thermal field.

Through the high‐temperature solid‐state synthesis, the as‐prepared Pr^3+^‐doped LiYO_2_ phosphors were confirmed to be a pure monoclinic phase (ICSD#50 992) with high crystallinity according to X‐ray powder diffraction (XRD) characterization (Figure S1a and Table S1, Supporting Information). In situ XRD measurement further revealed the occurrence of phase transition from monoclinic to tetragonal structure (ICSD#50 993) as the temperature increased from RT to 338 K (Figure 2b; Figure S1b, Supporting Information). This phase transition resulted in a drastic change of the vibrionic lattice mode related to the cation and its coordination polyhedron. In situ Raman spectra revealed the local symmetric change of [YO_6_] by the emergence of a new peak at 505 cm^−1^ and the vanishing of peaks at 136, 485, and 520 cm^−1^ (Figure S1c, Supporting Information).^[^ ^12^ ^]^ In addition, differential scanning calorimetric (DSC) measurements in a heating‐cooling cycle detected endothermic (enthalpy ≈ 6.61 J g^−1^) and exothermic (enthalpy ≈ 4.15 J g^−1^) peaks with a small thermal hysteresis, indicating reversible and first‐order nature of the phase transition (Figure S1d, Supporting Information).

As a result of the phase transition, the photoluminescence (PL) emission profile of Pr^3+^ varied substantially at ≈327 K upon the excitation into the 4*f* → 5*d* transition of Pr^3+^ at 280 nm (Figure 2c; Figure S2a,b, Supporting Information). In specific, the intensity ratio of red (^1^D_2_ → ^3^H_4_) and blue (^3^P_0,1_ → ^3^H_4_) emissions was thermally enhanced, accompanied by a noticeable blue shift of the ^1^D_2_ → ^3^H_4_ emission band. It is worth noting that the peak shift only occurred within the temperature range of 320–338 K, in good coincidence with the phase‐transition process (Figure S3, Supporting Information). This observation can be ascribed to short‐range structural alteration around Pr^3+^ ions during the thermally induced phase transition in LiYO_2_ that modulates the energy level splitting and radiative transition rates of *4f* levels (Figure S2c,d, Supporting Information).^[^ ^8a^ ^]^ Moreover, the decreased interionic distance of Y‐Y from 5.721 to 4.511 Å (*β* → *α*) favors the population at ^1^D_2_ relative to ^3^P_0,1_ by virtue of enhanced ^3^P_0_ + ^3^H_4_ → ^1^D_2_ + ^3^H_6_ cross relaxations.^[^ ^13^ ^]^ Due to the sharp increase in the red‐to‐blue emission intensity ratio near the phase‐transition temperature, we recorded an “S”‐shaped LIR curve against temperature (Figure 2d). Accordingly, a distinct switch in emission color from yellow to red was observed as the temperature increased across the phase‐transition point (Inset of Figure 2c; Figure S2e, Supporting Information). It is worth mentioning that a low doping level (≈0.25%) was set to minimize the interference from the concentration quenching (Figure S4, Supporting Information).^[^ ^14^ ^]^

The sensitive spectral response to minor thermal variation driven by phase transition provides an exciting opportunity for luminescence thermometry. Accordingly, the quantitative evaluation of thermometric performance was benchmarked by relative sensitivity (*S*_r_) and temperature resolution (*δT*). By plotting *S*_r_ against temperature, we found a rapid rise and decline of *S*_r_ within a temperature range near the phase‐transition point of LiYO_2_:Pr^3+^, registering a maximal value of 26.1% K^−1^ at 327 K (Figure 2e). It is worth noting that the identical *S_r_* values were obtained in three separate measurements, validating the reliability and accuracy of the as‐obtained high *S_r_* value (Figure S5, Supporting Information). The lowest detection limit *δT* was determined to be 0.008 K at 327 K in LiYO_2_:Pr^3+^ according to the standard deviation of LIR values obtained by recording PL spectra 200 times under identical conditions. Similar results were obtained using a charge‐coupled device (CCD) with a short acquisition time of 100 ms, confirming the reliability of the excellent temperature resolution (Figure S6, Supporting Information). It is worth noting that the excellent *δT* value mainly stemmed from the ultra‐high *S_r_* value and optimum measurement conditions with minimal background signals. On account of the high reversibility of the phase transition, the proposed LIR thermometry showed excellent repeatability over multiple heating‐cooling cycles (Figure 2f).

**Figure 3:**
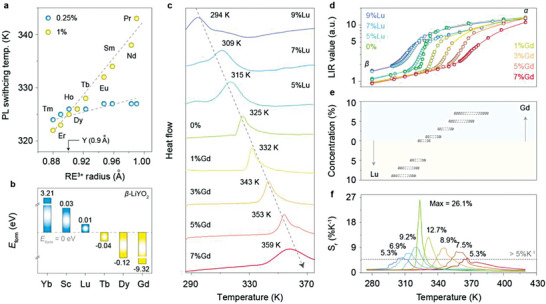
Controlling the phase‐transition temperature by doping. a) PL switching temperature of LiYO_2_:RE^3+^ (0.25%, 1%; RE = Pr, Nd, Sm, Eu, Tb, Dy, Ho, Er, and Tm) versus the ionic radius of rare‐earth dopants. b) The calculated formation energy (*E*_form_) of *β‐*LiYO_2_ doped with Yb^3+^, Sc^3+^, Lu^3+^, Tb^3+^, Dy^3+^, and Gd^3+^ ions, respectively. c) DSC curves of LiYO_2_:Pr (0.25%) co‐doped with different concentrations of Gd^3+^ (0–9%) or Lu^3+^ (0–7%). d) The calculated LIR value at different temperatures for all samples. e) Corresponding temperature range of the PL switching. f) The calculated *S*_r_ value at different temperatures for all samples.

Apart from Pr^3+^ ion, thermally induced changes in PL profiles, including the intensity ratio, shape, position, and Stark splitting number of emission bands, were also detected in other lanthanide ions (Nd^3+^, Sm^3+^, Eu^3+^, Tb^3+^, Dy^3+^, Ho^3+^, Er^3+^, and Tm^3+^; Figure S7, Supporting Information). These observations supported the dominant role of crystal phase and local structure in altering the luminescence property of lanthanide dopants. On top of that, we found that the temperature at which the PL switching occurred gradually shifted toward low temperature as the ionic radii of dopants constantly decreased (**Figure** **3**a). The dependence of PL switching temperature on the ionic radius is well manifested by increasing the doping concentration of lanthanide ions from 0.25% to 1% (Figure S8, Supporting Information). Furthermore, the full replacement of the Y^3+^ by smaller ions (Er^3+^, Tm^3+^, Yb^3+^, Lu^3+^, Sc^3+^) resulted in the formation of *α* phase at around RT due to a substantially low phase‐transition point (Figure S9, Supporting Information).^[^ ^10b^ ^]^

The shift of phase‐transition temperature can be ascribed to the change of structural stability induced by lanthanide dopants.^[^ ^8^, ^15^ ^]^ Due to the difference in cell volume between *β* and *α* phase (206.53 vs 204.03 Å^3^), the skeleton of *β* phase can collapse into *α* phase by doping smaller ions, while the introduction of a bigger one can well stabilize *β* structure. This assumption was validated by the dopant‐induced changes in the crystal formation energy based on the density functional theory (DFT) calculations (Figure 3b; Table S2, Supporting Information). Taken together, the structure of the *β* phase would become less stable as the ionic radii of dopants decrease, resulting in a lower phase‐transition temperature and, consequently, a lower PL switching temperature. These observations inspired us to establish an ultrasensitive thermometric approach at arbitrary temperatures by engineering the phase‐transition behavior.

As a proof‐of‐concept, optically inert Lu^3+^ and Gd^3+^ ions were introduced as co‐dopants due to their smaller (0.861 Å) and larger (0.938 Å) ionic radii than Y^3+^ (0.9 Å). In order to fine‐tune the phase‐transition temperature of LiYO_2_:Pr^3+^, the doping concentrations of Lu^3+^ and Gd^3+^ ions were carefully controlled. The successful doping of Lu^3+^ or Gd^3+^ ions into the lattice was confirmed by the Rietveld refinement analysis of the XRD patterns (Figure S10, Supporting Information). Specifically, the calculated cell volume gradually decreased or increased by increasing the doping content of Lu^3+^ or Gd^3+^ (Figure S11 and Tables S3 and S4, Supporting Information). Compositional analysis by energy‐dispersive X‐ray spectroscopy further revealed the uniform distribution of the lanthanide dopant elements within a single microparticle (Figure S12, Supporting Information).

The phase‐transition behavior of the co‐doped samples was next validated by DSC thermograms (Figure 3c). As expected, we detected a continuous shift of the phase‐transition point in the temperature range of 294–359 K by precisely controlling the dopant concentration of Lu^3+^ (0–9%) and Gd^3+^ (0–7%). Dopant‐induced tuning of phase‐transition temperature was further supported by the in situ XRD measurements and Raman spectroscopy (Figure S13, Supporting Information). As a result, we achieved progressive PL switching of Pr^3+^ ions as the temperature gradually increased (Figure 3d; Figure S14, Supporting Information). Notably, the temperature range of the PL switching matched well with that of the phase transition (Figure 3e; Table S5, Supporting Information). By extracting the spectral information of relevant samples near the phase‐transition points, constant ultra‐high thermal sensitivities (>5% K^−1^) and resolution (<0.03 K) can be achieved in a temperature range of 310–370 K (Figure 3f; Figure S15, Supporting Information). The obtained thermometric performance outperformed the existing luminescent thermometers in various configurations as far as we know, mainly stemming from the finely tunable phase‐transition characteristics of LiYO_2_ crystals (Table S6, Supporting Information). Note that the broadened phase‐transition temperature range and degraded maximum *S*_r_ in the co‐doped samples may be attributed to the dopant‐induced disorder in the domain structure.^[^ ^16^ ^]^

**Figure 4:**
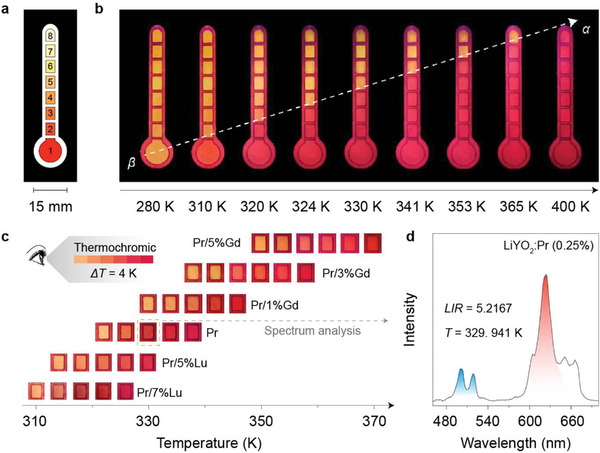
Phase‐transition‐driven colorimetric thermometry. a) Schematic of the CTI pattern composed of #1–8 samples: LiYO_2_:Pr^3+^/Lu^3+^ (0.25/9%, 7%, 5%), LiYO_2_:Pr^3+^ (0.25%), and LiYO_2_:Pr^3+^/Gd^3+^ (0.25/1%, 3%, 5%, 7%). b) Photographs of the CTI pattern at different temperatures under the excitation of a handheld 275 nm lamp. c) The delicate variations of emission color within the temperature range of phase transition for representative #2–7 samples: LiYO_2_:Pr^3+^/Lu^3+^ (0.25/7%, 5%), LiYO_2_:Pr^3+^ (0.25%), and LiYO_2_:Pr^3+^/Gd^3+^ (0.25/1%, 3%, 5%). The temperature interval was 4 K. d) PL spectrum of LiYO_2_:Pr^3+^ (0.25%) recorded at the position indicated by a dashed box in (c) The LIR and temperature were calculated to be 5.2167 and 329.941 K, respectively.

The successful control of phase‐transition behavior greatly expands the practical utility of phase‐transition‐driven thermometry. Significantly, different LiYO_2_ crystals together can provide sequential thermochromic PL switching, enabling convenient temperature indication and quantification. In a specific demonstration, we devised a colorimetric temperature indicator (CTI) by patterning a set of LiYO_2_:Pr^3+^/Lu^3+^ (0.25/5–17%), LiYO_2_:Pr^3+^ (0.25%), and LiYO_2_:Pr^3+^/Gd^3+^ (0.25/3–7%) crystals on an aluminum alloy substrate (**Figure** **4**a). Under UV excitation at 275 nm, the whole pattern displayed uniform yellow emission at around 280 K, resulting from a consistent *β* phase in all samples. As the temperature gradually increased, individual segments of the pattern turned pure red in sequence due to progressive phase transition in the samples (Figure 4b). Accordingly, the color‐switching progress along the sample sequence provided a direct temperature indication discernible by the naked eye, with a temperature resolution of ≈4 K. Notably, more accurate temperature values can be obtained by examining the exact color or emission spectrum of the sample in the phase‐transition process (Figure 4c,d). On a separate note, the programmable PL color switching in the LiYO_2_ crystals can also enable the storage of temperate‐specific optical codes, holding great promise for high‐security data protection (Figure S16, Supporting Information).

**Figure 5:**
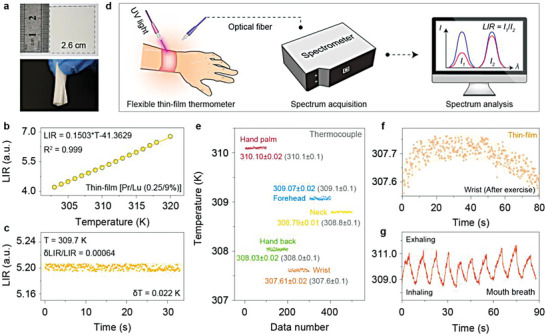
Wearable thin‐film thermometer for healthcare monitoring. a) Photographs of the as‐fabricated (top panel) and rolled up (bottom panel) thin‐film thermometer with a side length of 2.6 cm and a thickness of 0.05 cm. b) The calculated LIR value of the as‐fabricated thin‐film sensor versus the temperature in the 303–320 K range. c) Fluctuation of LIR value at a constant temperature of 309.7 K during the continuous collection of PL spectra (acquisition time: 0.1 s). d) Schematic diagram of the experimental setup for real‐time body temperature detection. e) Measured temperatures of the human body parts by the wearable sensor and a thermocouple (grey values) fitting on the wrist, forehead, neck, palm, and back of the hand, respectively. Note that the temperature data were measured continuously through PL or a thermocouple, which were presented with the corresponding errors. Real‐time monitoring of f) wrist temperatures after exercise and g) breath temperatures of a volunteer by the thin‐film thermometer.

Owing to the ultra‐high thermal sensitivity in the customizable operating range, single‐component LiYO_2_ crystals also excel in particular application scenarios. For example, maximum sensitivity in the physiological temperature range (≈310 K) can be obtained by employing LiYO_2_:Pr^3+^/Lu^3+^ (0.25/9%) crystals for precise body temperature monitoring. As a proof‐of‐concept, we fabricated a flexible thin‐film thermometer by embedding the powder samples into a polydimethylsiloxane (PDMS) substrate. Notably, the as‐prepared thin film featured good mechanical stability and flexibility, permitting repeated rolling without noticeable structural failure (**Figure** **5**a). We measured the LIR values of the thin film against temperature in the 303–320 K range as the calibration curve, which revealed a perfect linear fit (*LIR* = 0.1503*T – 41.3629; Figure 5b). A small standard deviation of the LIR signals was observed at a constant temperature of 309.7 K during the continuous collection of PL spectra over 300 times. This remarkable stability of LIR signals rendered a high‐temperature resolution of up to around 0.022 K, in close resemblance to that of the powder samples (Figure 5c; Figures S17 and S18, Supporting Information). The performance well satisfies the requirements of healthcare applications (*δT* ≈ 0.1 K in the range of 310–312 K).^[^ ^17a^ ^]^ Moreover, the thin‐film sensor provided a response time as fast as the thermocouple in two heating‐cooling cycles (Figure S19, Supporting Information).

In an illustrative design, the optical signals of the thin film fitted on the human body were collected by a CCD under UV excitation. Based on the calibration curve of LIR with temperature, these PL data were analyzed in real‐time and converted into temperature information using computer software (Figure 5d). Remarkably, the accurate temperatures of some important parts of the human body, such as the wrist, forehead, neck, palm, fingers, and back of the hand, were instantly detected by this wearable sensor (Figure 5e). Notably, the as‐fabricated wearable sensor featured a higher resolution in comparison with a thermocouple (≈0.1 K), enabling more precise thermometry for healthcare applications (Figures S20 and S21, Supporting Information). Meanwhile, the heat absorption and release accompanied by human activities, such as exercise and breath, can be precisely captured with fast response and good repeatability (Figure 5f,g).^[^ ^17b,c^ ^]^ These results demonstrated the superiorities of the phase‐transition‐driven LIR thermometers as intelligent wearable devices for the timely assessment of physiological parameters related to cardiovascular health.

## 3 Conclusion

In summary, we have achieved programmable luminescence switching in LiYO_2_:Pr^3+^ crystals under thermal stimuli, based on rational control over *β* → *α* phase transition of the host lattice. We highlighted an efficient tactic for precisely tuning the phase‐transition temperature over a wide range of 294–359 K by Lu^3+^ and Gd^3+^ substitution. As a result, rapid luminescence switching from yellow to pure red is realized at prescribed transition temperatures. This sensitive spectral response to minor thermal variation can be harnessed for colorimetric thermometry with superior sensitivity and temperature resolution (*S*_r_ = 26.1% K^−1^, *δT* = 0.008 K) in a temperature range of 310–370 K. These findings may inspire new ideas in designing innovative stimulus‐response optical materials for healthcare‐oriented intelligent wearable devices.

## 4 Experimental Section

### Materials

Li_2_CO_3_ (Aladdin, 99.99%), Y_2_O_3_ (Aladdin, 99.99%), Pr_6_O_11_ (Aladdin, 99.99%), Lu_2_O_3_ (Aladdin, 99.99%), Gd_2_O_3_ (Aladdin, 99.99%), Sc_2_O_3_ (Aladdin, 99.99%), Nd_2_O_3_ (Aladdin, 99.99%), Sm_2_O_3_ (Aladdin, 99.99%), Eu_2_O_3_ (Aladdin, 99.99%), Tb_4_O_7_ (Aladdin, 99.99%), Dy_2_O_3_ (Aladdin, 99.99%), Ho_2_O_3_ (Aladdin, 99.99%), Er_2_O_3_ (Aladdin, 99.99%), Tm_2_O_3_ (Aladdin, 99.99%), and Yb_2_O_3_ (Aladdin, 99.99%), were all purchased and used as received.

### Preparation of LiYO_2_:RE^3+^ Phosphors

Rare‐earth ions (RE^3+^) doped LiYO_2_ powder samples were all prepared by a high‐temperature solid‐state reaction. First, raw materials were weighed based on the nominal composition of LiY_1‐_ *_x_* O_2_:*x*RE^3+^ and then ground thoroughly in an agate mortar. Then, the mixtures were directly calcined at 1100 °C for 5 h (heating rate: 5°C min^−1^) under a protection gas flow of 90% N_2_ & 10% H_2_. After naturally cooling down to room temperature (RT), the resulting samples were homogeneously ground into fine powders for subsequent characterizations. The synthesis procedures of LiREO_2_ (RE = Tb, Dy, Ho, Er, Tm, Yb, Lu, Sc) samples were similar, except that Y_2_O_3_ was replaced with corresponding RE_2_O_3_ in the first step.

### Preparation of Flexible Thin‐Film Thermometer

The thin‐film thermometer was composed of transparent polydimethylsiloxane (PDMS), curing agent, and LiYO_2_:Pr^3+^/Lu^3+^ (0.25/9%) sample in the weight ratio of 10:1:2. First, the curing agent was premixed with SYLGARD 184 silicone elastomer base, to which the sample was added under vigorous stirring. Subsequently, the mixture was transferred into a petri dish (diameter: 4 cm) for casting, followed by the degassing treatment in a vacuum oven to remove air bubbles. Finally, the uncured film was dried at 70 °C for 2 h to get the PDMS thin film (side length: 2.6 cm, thickness: 0.5 mm)

### Characterization

X‐ray powder diffraction (XRD) patterns at different temperatures were acquired by a Bruker D8 Advance powder diffractometer with Cu‐Kα radiation, and the crystal structure refinements were analyzed using the General Structure Analysis System (GASA‐II). The microstructure of the samples was characterized by a Novanano‐450 field scanning electron microscopy (SEM). Differential scanning calorimetric (DSC) curves of the samples were measured by a NETZSCH 204 HP synchronous thermal analyzer (heating rate: 5 K min^−1^) in an N_2_ atmosphere. Raman spectra were collected by HR Evolution confocal Raman microscope at different temperatures. The optical property at different temperatures, including photoluminescence emission (PL) and excitation (PLE) spectra, was measured by a Horiba FL3 fluorescence spectrometer equipped with a 450 W xenon lamp as the excitation source. The luminescence images of the samples at different temperatures were taken by an HONOR 20 phone fixed on a holder. To evaluate the performance of the wearable thin‐film thermometer, a real‐time temperature testing system using a 275 nm lamp and an Ocean Optical QEpro fiber spectrometer as the excitation source and detector, respectively, were built. The optical signals of the thin film could be continuously collected by a fiber‐coupled spectrometer (acquisition time: 0.1 s). At the same time, these PL data could be analyzed by an as‐edited packaged software on the computer and converted into LIR and temperature information in real‐time. It should be noted that the distance and angle between the thin film and lamp or detector were kept consistent in different testing scenes. A K‐type thermocouple probe (Tasi, TA8113) was employed to measure the temperature changes of water as the reference data.

### Computation Details

All the theoretical calculations were performed by Vienna Ab Initio Simulation Package (VASP).^[^ ^18^ ^]^ The formation energy was calculated by density functional theory (DFT) in the form of generalized gradient approximation (GGA) Perdew–Burke–Ernzerhof (PBE).^[^ ^19^ ^]^ A Monkhorst–Pack 2 × 4 × 5 k mesh was used as Brillouin zone, and the kinetic energy cutoff and self‐consistent field (SCF) were set as 500 eV and 10^−5^ eV per atom, respectively.

## Conflict of Interest

The authors declare no conflict of interest.

## Supporting information

Supporting Information

## Data Availability

The data that support the findings of this study are available in the supplementary material of this article.

## References

[1] a) J. Zhou , B. Del Rosal , D. Jaque , S. Uchiyama , D. Jin , Nat. Methods. 2020, 17, 967; 32989319 10.1038/s41592-020-0957-y

[2] a) M. Xu , X. Zou , Q. Su , W. Yuan , C. Cao , Q. Wang , X. Zhu , W. Feng , F. Li , Nat. Commun. 2018, 9, 2698; 30002372 10.1038/s41467-018-05160-1 PMC6043590

[3] a) E. C. Ximendes , U. Rocha , T. O. Sales , N. Fernández , F. Sanz‐Rodríguez , I. R. Martín , C. Jacinto , D. Jaque , Adv. Funct. Mater. 2017, 27, 1702249;

[4] a) H. Suo , X. Zhao , Z. Zhang , Y. Wang , J. Sun , M. Jin , C. Guo , Laser Photonics Rev. 2021, 15, 2000319;

[5] a) A. Bednarkiewicz , J. Drabik , K. Trejgis , D. Jaque , E. Ximendes , L. Marciniak , Appl. Phys. Rev. 2021, 8, 011317;

[6] a) B. Zheng , J. Fan , B. Chen , X. Qin , J. Wang , F. Wang , R. Deng , X. Liu , Chem. Rev. 2022, 122, 5519; 34989556 10.1021/acs.chemrev.1c00644

[7] a) H. Dong , L.‐D. Sun , C.‐H. Yan , Chem. Soc. Rev. 2015, 44, 1608; 25242465 10.1039/c4cs00188e

[8] a) H. Dong , L.‐D. Sun , C.‐H. Yan , J. Am. Chem. Soc. 2021, 143, 20546; 34865480 10.1021/jacs.1c10425

[9] a) D. Ananias , F. A. A. Paz , D. S. Yufit , L. D. Carlos , J. Rocha , J. Am. Chem. Soc. 2015, 137, 3051; 25664963 10.1021/ja512745y

[10] a) M. D. Faucher , O. K. Moune , M.‐G. Alves , B. Piriou , P. Sciau , M. Pham‐Thi , J. Solid State Chem. 1996, 121, 457;

[11] a) X. Zhou , L. Ning , J. Qiao , Y. Zhao , P. Xiong , Z. Xia , Nat. Commun. 2022, 13, 7589; 36481731 10.1038/s41467-022-35366-3 PMC9732309

[12] L. Marciniak , W. M. Piotrowski , M. Drozd , V. Kinzhybalo , A. Bednarkiewicz , M. Dramicanin , Adv. Opt. Mater. 2022, 10, 2102856.

[13] J. Stefanska , L. Marciniak , Adv. Photonics Res. 2021, 2, 2100070.

[14] L. Liang , X. Qin , K. Zheng , X. Liu , Acc. Chem. Res. 2019, 52, 228. 30557000 10.1021/acs.accounts.8b00469

[15] M. Hong , Z.‐G. Chen , L. Yang , Y.‐C. Zou , M. S. Dargusch , H. Wang , J. Zou , Adv. Mater. 2018, 30, 1705942. 10.1002/adma.201705942 29349887

[16] A. Shukla , R. N. P. Choudhary , A. K. Thakur , D. K. Pradhan , Physica B Condens. Matter. 2010, 405, 99.

[17] a) Q. Li , L.‐N. Zhang , X.‐M. Tao , X. Ding , Adv. Healthcare Mater. 2017, 6, 1601371; 10.1002/adhm.201601371 28547895

[18] M. Ernzerhof , G. E. Scuseria , J. Chem. Phys. 1999, 110, 5029.

[19] M. Liu , C.‐K. Duan , P. A. Tanner , C.‐G. Ma , X. Wei , M. Yin , Phys. Rev. B 2022, 105, 195137.

