# Supplementary material for: Ultrasensitive Colorimetric Luminescence Thermometry by Progressive Phase Transition
Source: Adv Sci (Weinh). 2023 Dec 11;11(7):2305241. doi: 10.1002/advs.202305241 (PMC10870082; doi:10.1002/advs.202305241)
Supplement: Supplementary file 1 — Supporting Information [file ADVS-11-2305241-s001.pdf]

ADVANCED SCIENCE
Open Access

Supporting Information

for *Adv. Sci.*, DOI 10.1002/advs.202305241

Ultrasensitive Colorimetric Luminescence Thermometry by Progressive Phase Transition

Hao Suo, Dongxu Guo, Peihang Zhao, Xin Zhang, Yu Wang, Weilin Zheng, Panlai Li, Tao Yin, Li Guan, Zhijun Wang* and Feng Wang**

Supporting Information

Ultrasensitive Colorimetric Luminescence Thermometry by Progressive Phase Transition

Hao Suo#, Dongxu Guo #, Peihang Zhao, Xin Zhang, Yu Wang, Weilin Zheng, Panlai Li, Tao Yin, Li Guan, Zhijun Wang*, Feng Wang**

Address correspondence to E-mail: suo@hbu.edu.cn, wangzj1998@126.com, and fwang24@cityu.edu.hk

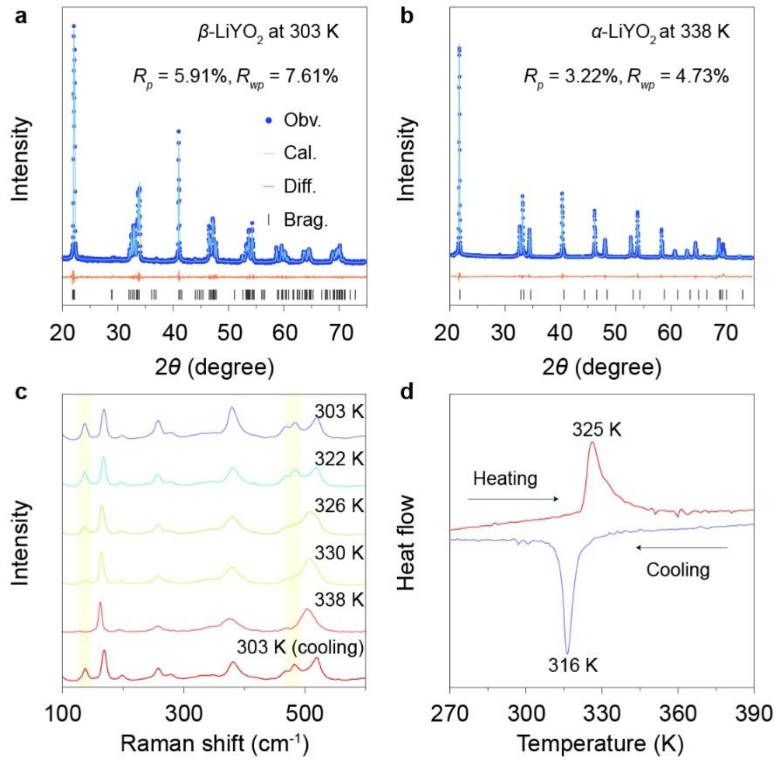

Figure S1. Rietveld refinements of XRD patterns recorded at **a)** 303 and **b)** 338 K for as-prepared $LiYO_2:Pr^{3+}$ (0.25%) crystals. It can be found that monoclinic β and tetragonal α phase was obtained with high purity and crystallinity at 303 and 338 K, respectively. The calculated crystallographic structural parameters are summarized in **Table S1**. **c)** In-situ Raman spectra of $LiYO_2:Pr^{3+}$ (0.25%) at different temperatures. In specific, the peaks at 136, 484, and 520 cm^{-1} of β-$LiYO_2$ crystal are attributed to the translational modes of [YO_6] and [LiO_6], bending modes of [YO_6], and O-Y-O stretching mode, respectively. At elevated temperatures, the new peak at 505 cm^{-1} of α-$LiYO_2$ crystal is assigned to the bending modes of [YO_6]. **d)** DSC curves of the sample upon heating and cooling.

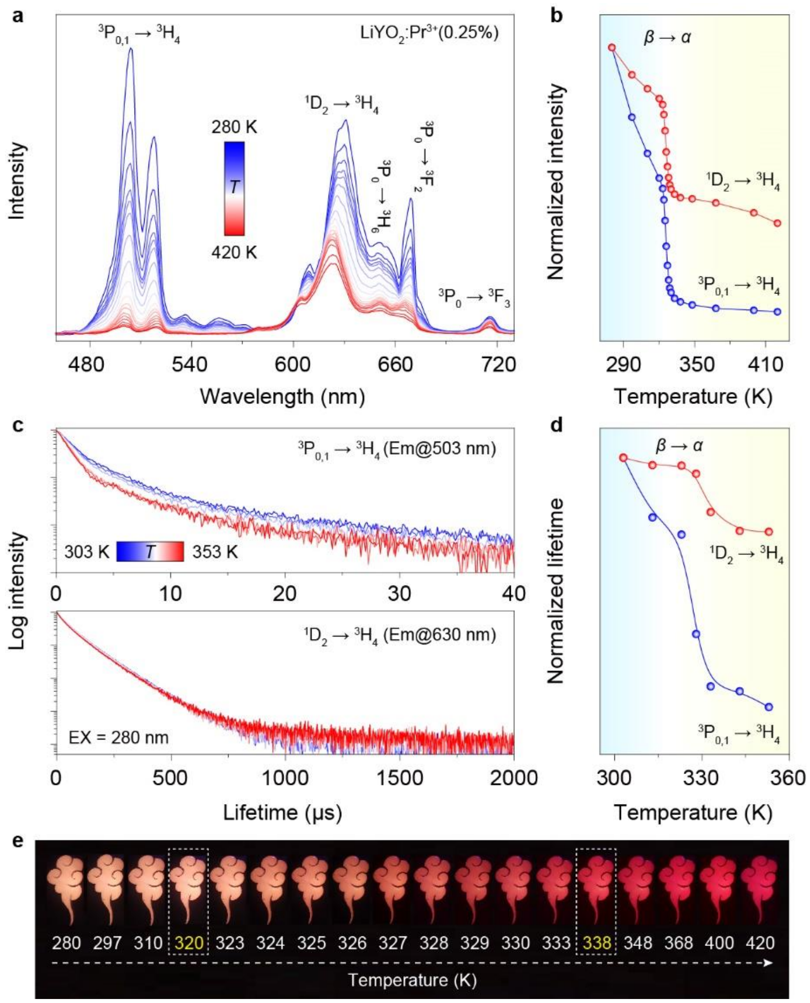

Figure S2. a) Temperature-dependent PL spectra of $LiYO_2$: Pr^{3+} (0.25%) crystals under 280 nm excitation in the 280–420 K range. **b)** Normalized emission intensity of $^1D_2 \rightarrow {}^3H_4$ and $^3P_{0,1} \rightarrow {}^3H_4$ transitions as a function of temperature. Note that the emission band in the 600–645 nm range gradually shifted toward the longer wavelength, while the $^3P_{0,1} \rightarrow {}^3H_4$ transitions were hardly shifted by temperature. The results further suggested that the emission band in the 600–645 nm range is mainly attributed to the $^1D_2 \rightarrow {}^3H_4$ rather than $^3P_0 \rightarrow {}^3H_6$ transition. **c)** Temperature-dependent decay curves of the $^3P_{0,1} \rightarrow {}^3H_4$ (503 nm) and $^1D_2 \rightarrow {}^3H_4$ (630 nm) transitions under 280 nm excitation. **d)** Normalized lifetimes of the two transitions as a function of temperature. **e)** The luminescence images of the sample in an "auspicious clouds" pattern within the temperature range of 280–420 K. The white dashed boxes indicate the onset and completion temperatures of the PL switching process.

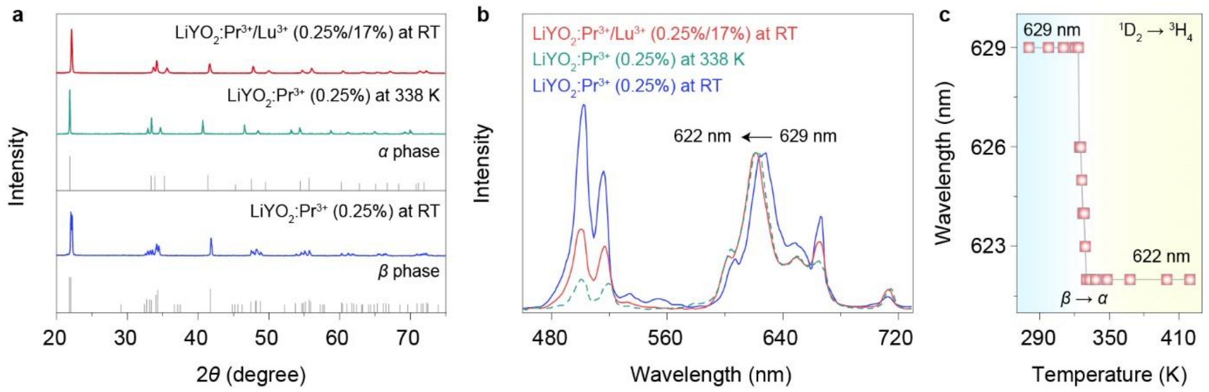

Figure S3. a) XRD patterns and **b)** PL spectra (λ_{ex} = 280 nm) of $LiYO_2$: Pr^{3+}/Lu^{3+} (0.25%/17%) at RT, $LiYO_2$: Pr^{3+} (0.25%) at RT and 338 K. **c)** Peak wavelength of $^1D_2 \rightarrow {}^3H_4$ emission as a function of temperature. Single-phased α-$LiYO_2$:Pr^{3+} with high purity at room temperature was successfully prepared by co-doping with 17% Lu^{3+} ions. Notably, the peak wavelength of $^1D_2 \rightarrow {}^3H_4$ emission in the α phase (~ 622 nm) was shorter than that in the β phase (~ 629 nm) at room temperature, identical to that in $LiYO_2$ at 338 K (~ 622 nm), ruling out the contribution of temperature alone to the blue shift of Pr^{3+} emission. Moreover, the blue shift of emission only occurred within the temperature range of 320–338 K, in good coincidence with the phase-transition process. Such correlation provided strong evidence that the observed blue shift in the emission spectra was ascribed to the thermal-induced phase transition in $LiYO_2$.

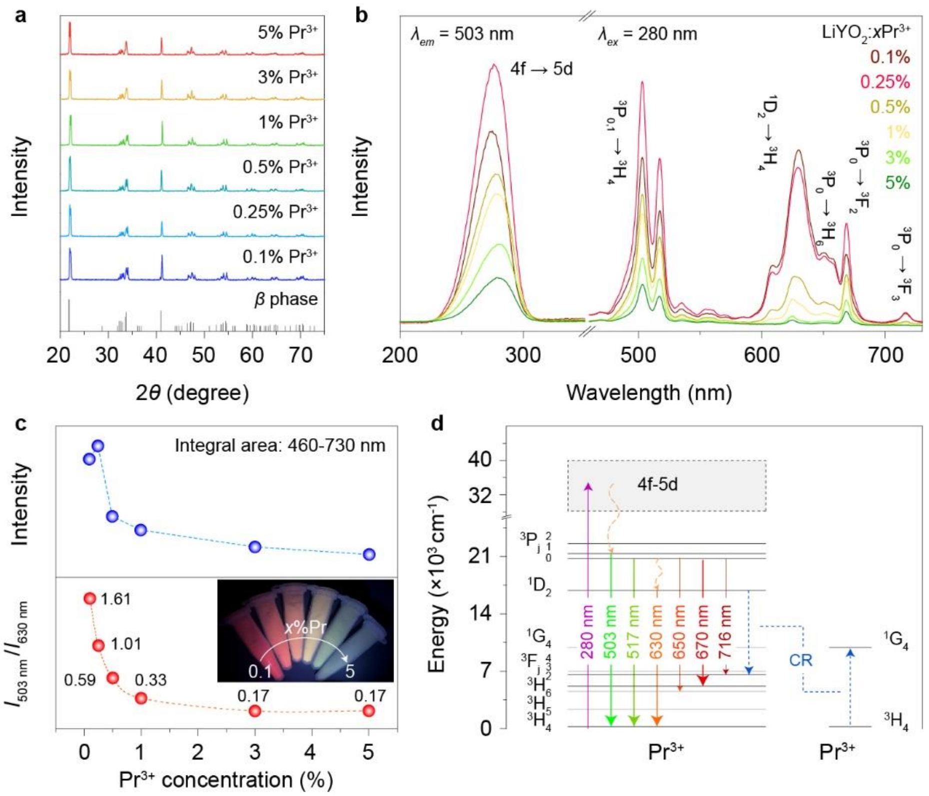

Figure S4. a) XRD patterns of $LiYO_2$: Pr^{3+} (0.1-5%) crystals. All these samples were confirmed to be pure monoclinic β phase $LiYO_2$, revealing the successful doping of Pr^{3+} into the host. **b)** PLE (λ_{em} = 503 nm) and PL spectra (λ_{ex} = 280 nm) of $LiYO_2$: Pr^{3+} (0.1-5%) crystals. **c)** Integral overall intensity and intensity ratio of $I_{503\ nm}/I_{630\ nm}$ ($I_{503\ nm}$: 490-520 nm, $I_{630\ nm}$: 600-645 nm) as a function of Pr^{3+} concentration. Inset shows the luminescence image of the samples under the excitation of a handheld 275 nm lamp. **d)** Schematic energy level diagram of Pr^{3+} ions with possible transitions marked. Notably, the emission intensity of Pr^{3+} first increased with the doping concentration, and then sharply decreased after reaching the optimal value of 0.25% due to the concentration quenching effect. Meanwhile, the emission band in the 600–645 nm range gradually declined in comparison with that of $^3P_{0,1} \rightarrow {}^3H_4$ at high Pr^{3+} doping levels, resulting in the color variation from orange-red to pure green. This phenomenon was ascribed to the increased probability of cross relaxation (CR) process ($^1D_2 + {}^3H_4 \rightarrow {}^3F_{3,4} + {}^1G_4$) that depopulates the $^1D_2$ level. Therefore, we determined an optimal Pr^{3+} concentration of 0.25% to avoid the serious concentration quenching effect. The above results indicated that the emission band in the 600–645 nm range under 280 nm excitation is mainly attributed to the $^1D_2 \rightarrow {}^3H_4$ rather than $^3P_0 \rightarrow {}^3H_6$ transition.

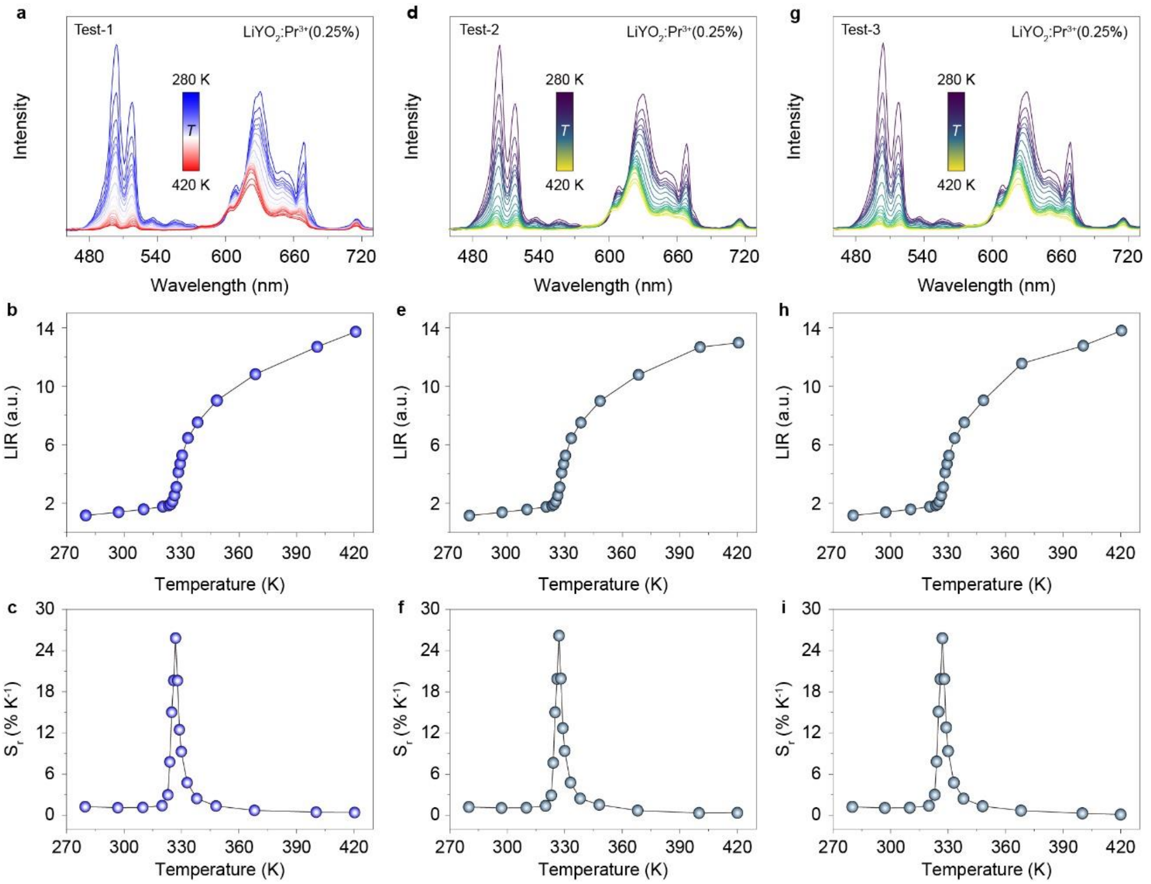

Figure S5. Determination of relative sensitivity under repeated measurements. As an important parameter for evaluating thermometric performance, relative sensitivity (S_r) is defined as the relative change rate of intensity ratio (*LIR*) associated with temperature (*T*): [1]

$$S_r = \frac{1}{LIR} \times \frac{dLIR}{dT} \quad \text{(Eq S1)}$$

According to the above equation, the calculated S_r value rapidly increased and then declined within a temperature range near the phase transition point of $LiYO_2:Pr^{3+}$, demonstrating a maximal value of 26.1% K^{-1} at 327 K. Notably, identical S_r values were obtained in three separate measurements, validating the reliability and accuracy of the as-obtained high S_r value.

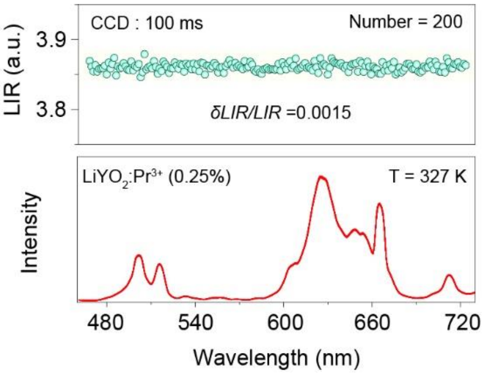

Figure S6. Normalized PL spectra measured for 200 times and the corresponding fluctuation of LIR values and calculated δT values using a CCD. Temperature resolution or uncertainty (δT) describes the smallest temperature change resolvable by the thermometer in a given measurement, which is widely expressed as: [2]

$$\delta T = \frac{1}{S_r} \times \frac{\delta LIR}{LIR} \quad \text{(Eq S2)}$$

where $\delta LIR/LIR$ is the relative uncertainty in determining the LIR value (depending on the signal-to-noise ratio and estimated from the errors in LIR) and S_r is the relative sensitivity at the relevant temperature. The results further confirmed the reliability of the high temperature resolution, where similar $\delta LIR/LIR$ values were obtained by recording the PL spectra up to 200 times using a PMT with an acquisition time of 60 s and a CCD with a short acquisition time of 100 ms.

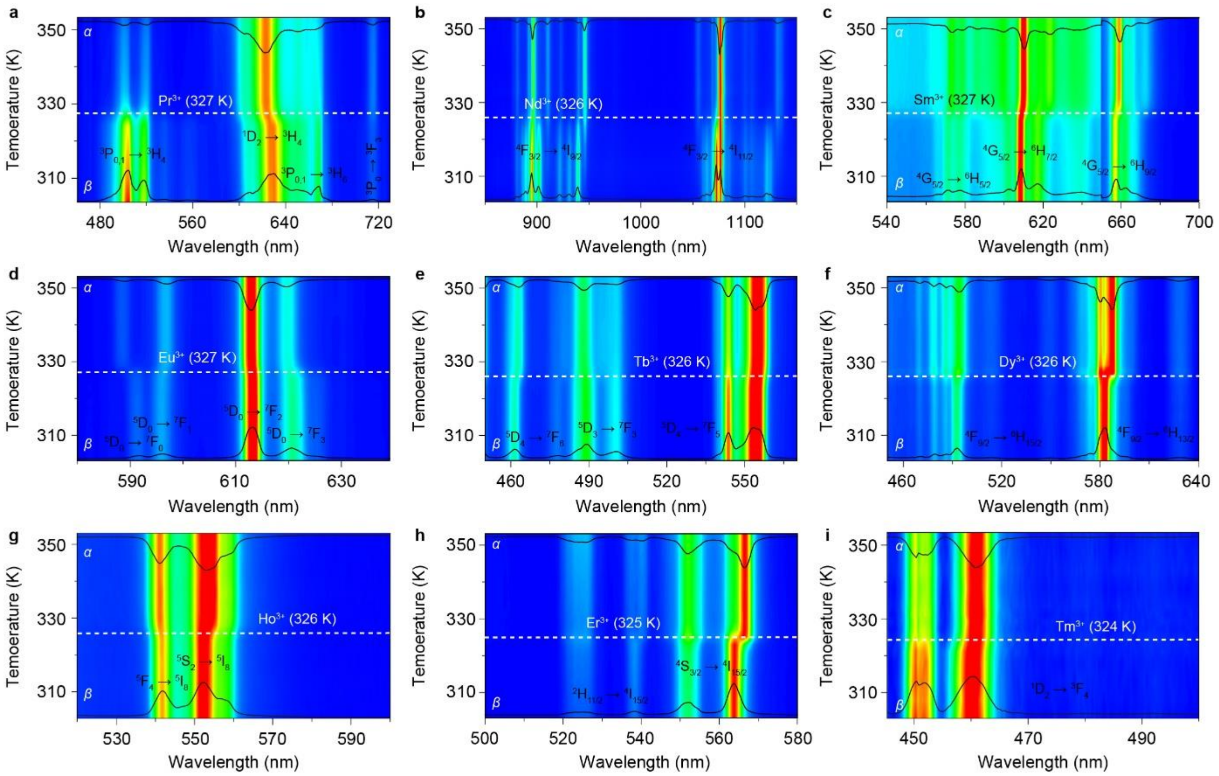

Figure S7. Normalized PL spectral mapping of $LiYO_2$ crystals doped with 0.25% of **a)** Pr^{3+}, **b)** Nd^{3+}, **c)** Sm^{3+}, **d)** Eu^{3+}, **e)** Tb^{3+}, **f)** Dy^{3+}, **g)** Ho^{3+}, **h)** Er^{3+}, and **i)** Tm^{3+} as a function of temperature in the 303–353 K range.

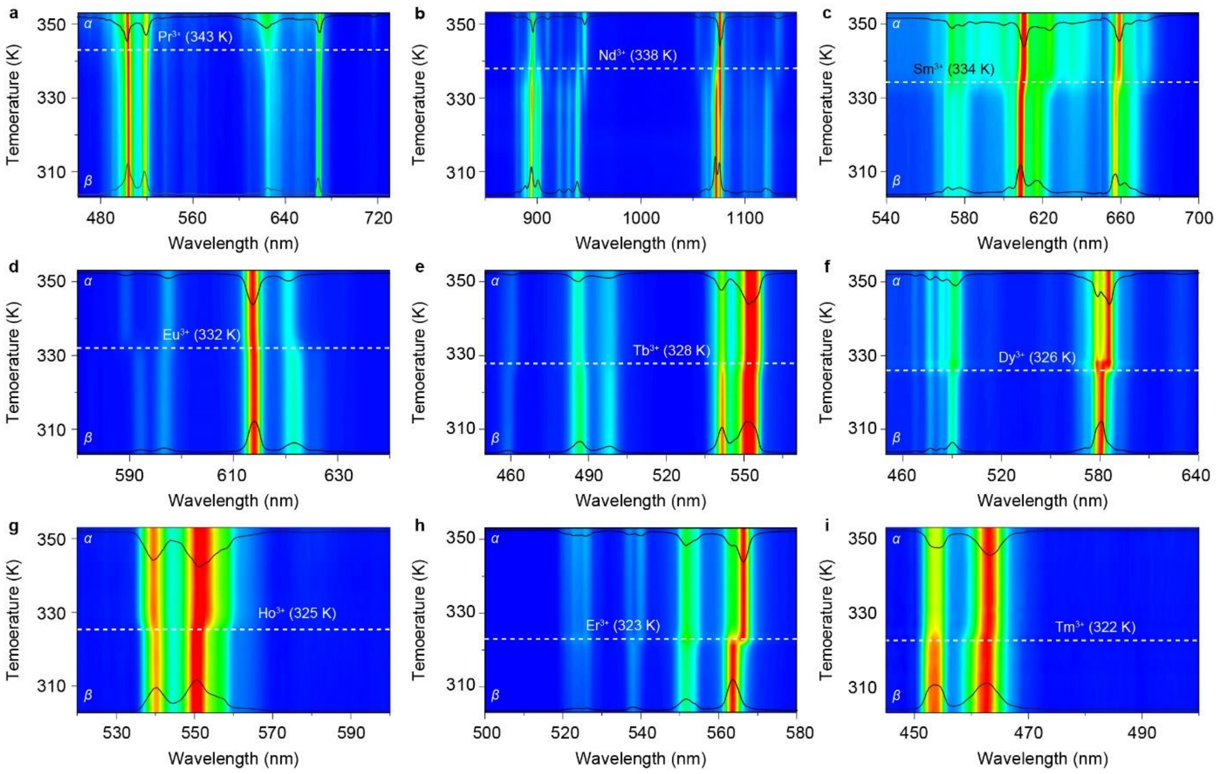

Figure S8. Normalized PL spectral mapping of $LiYO_2$ crystals doped with 1% of **a)** Pr^{3+}, **b)** Nd^{3+}, **c)** Sm^{3+}, **d)** Eu^{3+}, **e)** Tb^{3+}, **f)** Dy^{3+}, **g)** Ho^{3+}, **h)** Er^{3+}, and **i)** Tm^{3+} as a function of temperature in the 303–353 K range.

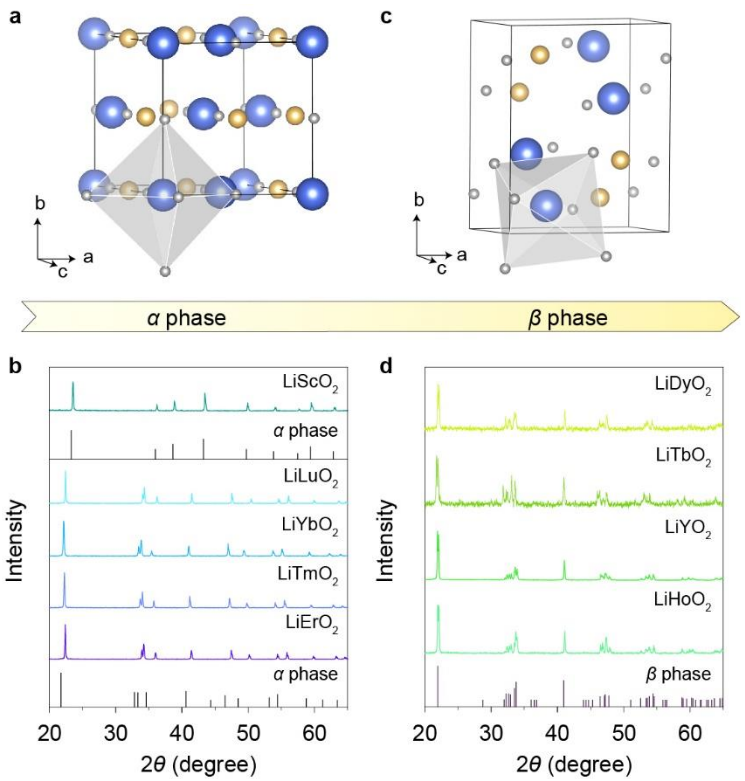

Figure S9. XRD patterns and the schematic crystal structure of **a-b**) tetragonal *α* phase $LiREO_2$ samples (RE = Sc, Lu, Yb, Tm, Er), **c-d)** monoclinic *β* phase samples (RE = Ho, Y, Dy, Tb).

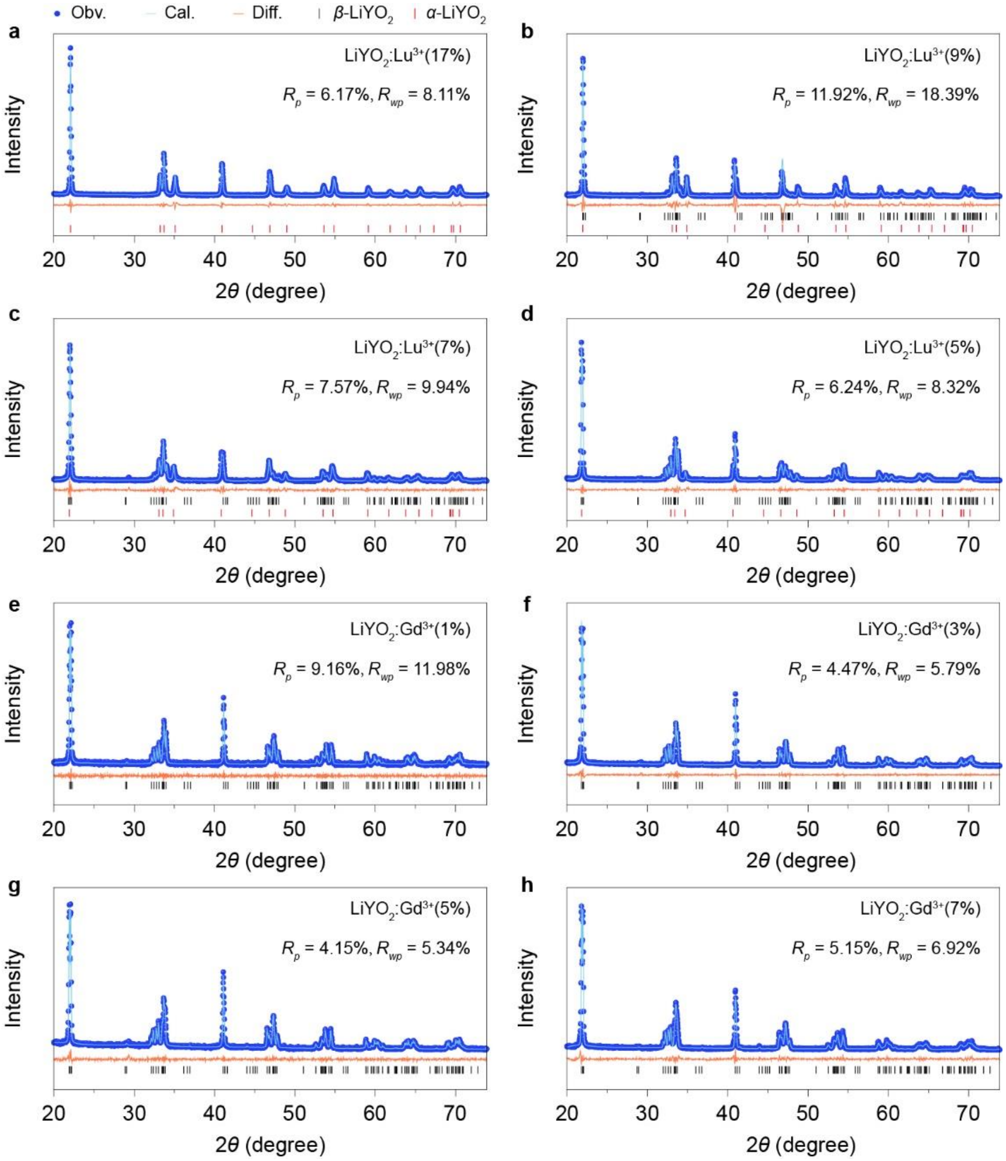

Figure S10. Rietveld refinements of XRD patterns for as-prepared $LiYO_2:Pr^{3+}$ (0.25%) co-doped with different concentrations of Gd^{3+} (1-7%) or Lu^{3+} (5-17%). The calculated crystallographic structural parameters are summarized in **Table S3-S4**.

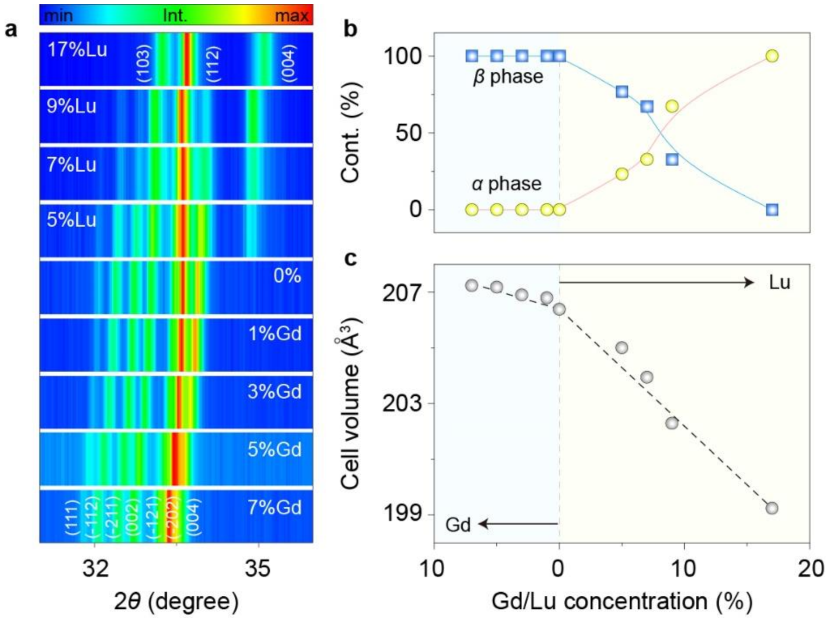

Figure S11. a) XRD patterns of $LiYO_2$:Pr^{3+} (0.25%) co-doped with different concentrations of Gd^{3+} or Lu^{3+} in the 31–36 degree range. **b)** The relative content of α-$LiYO_2$ in β-$LiYO_2$ and **c)** volume of the unit cell as a function of Gd^{3+} or Lu^{3+} concentration.

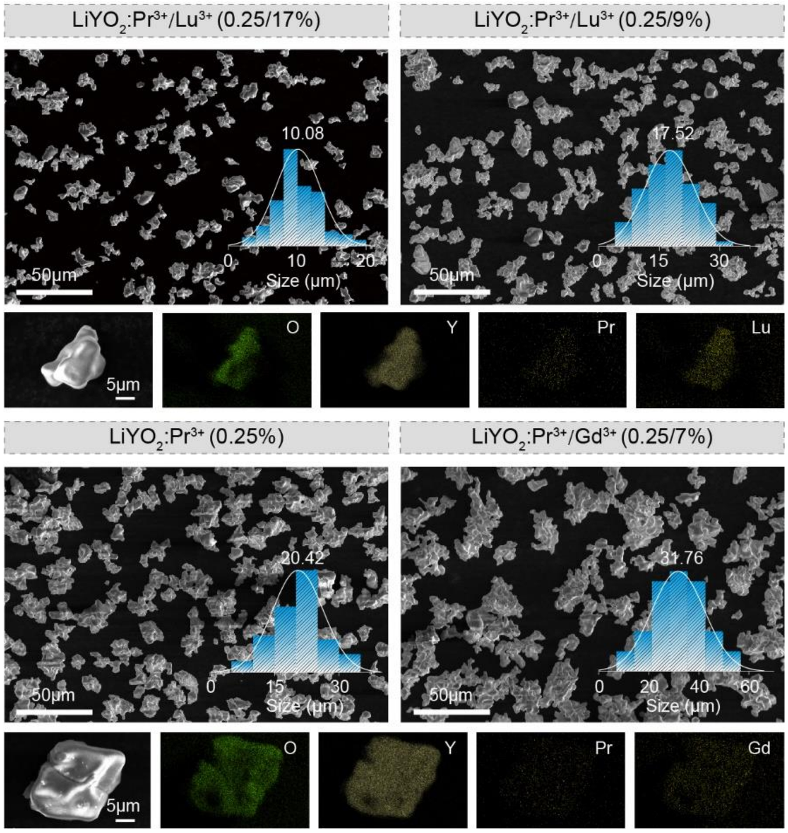

Figure S12. SEM images of $LiYO_2:Pr^{3+}$ (0.25%), $LiYO_2:Pr^{3+}/Gd^{3+}$ (0.25/7%), and $LiYO_2:Pr^{3+}/Lu^{3+}$ (0.25/17% and 0.25/9%), along with the element mapping of $LiYO_2:Pr^{3+}/Lu^{3+}$ (0.25%/9%) and $LiYO_2:Pr^{3+}/Gd^{3+}$ (0.25/7%). Inset shows the number-average size distribution of the as-prepared samples. Notably, all samples revealed similar irregular morphology with average sizes of around 10.08, 17.52, 20.42, and 31.76 μm for Pr^{3+}/Lu^{3+} (0.25/17%), Pr^{3+}/Lu^{3+} (0.25/9%), Pr^{3+} (0.25%), and Pr^{3+}/Gd^{3+} (0.25/7%) doped $LiYO_2$, respectively.

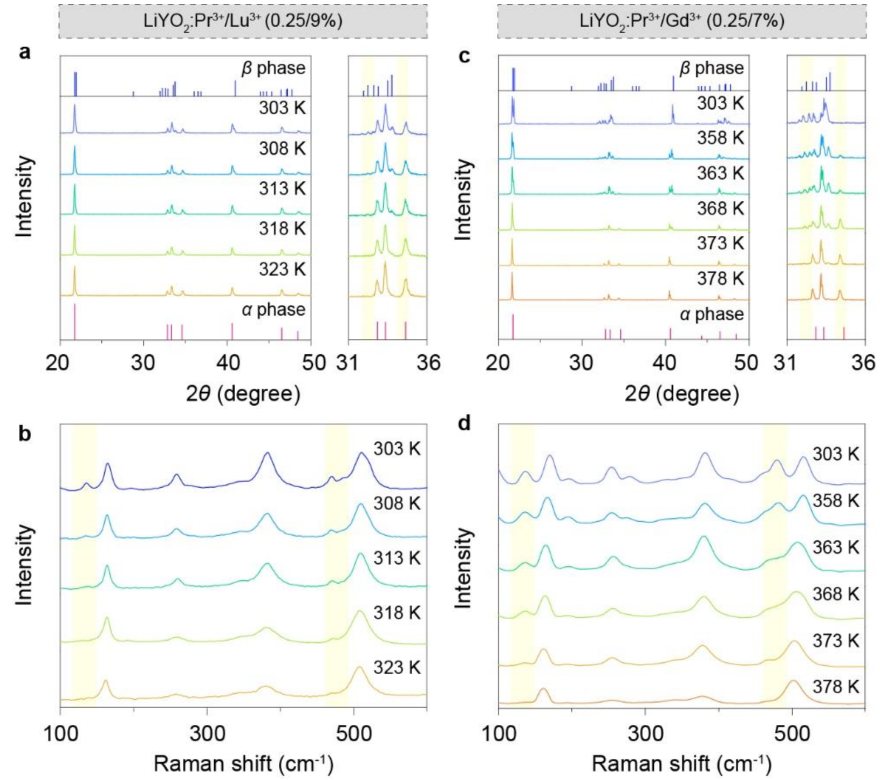

Figure S13. In-situ XRD patterns and Raman spectra of **a-b)** $LiYO_2$:Pr^{3+}/Lu^{3+} (0.25/9%) and **c-d)** $LiYO_2$:Pr^{3+}/Gd^{3+} (0.25/7%) at different temperatures. The results indicated the occurrence of phase transition from β to α structure in both samples. In specific, phase transition started below RT and finished at around 318 K after co-doping with 9%Lu^{3+} ions. In comparison, the introduction of 7%Gd^{3+} ions enhanced the start and ending temperature required for phase transition to around 358 K and 378 K.

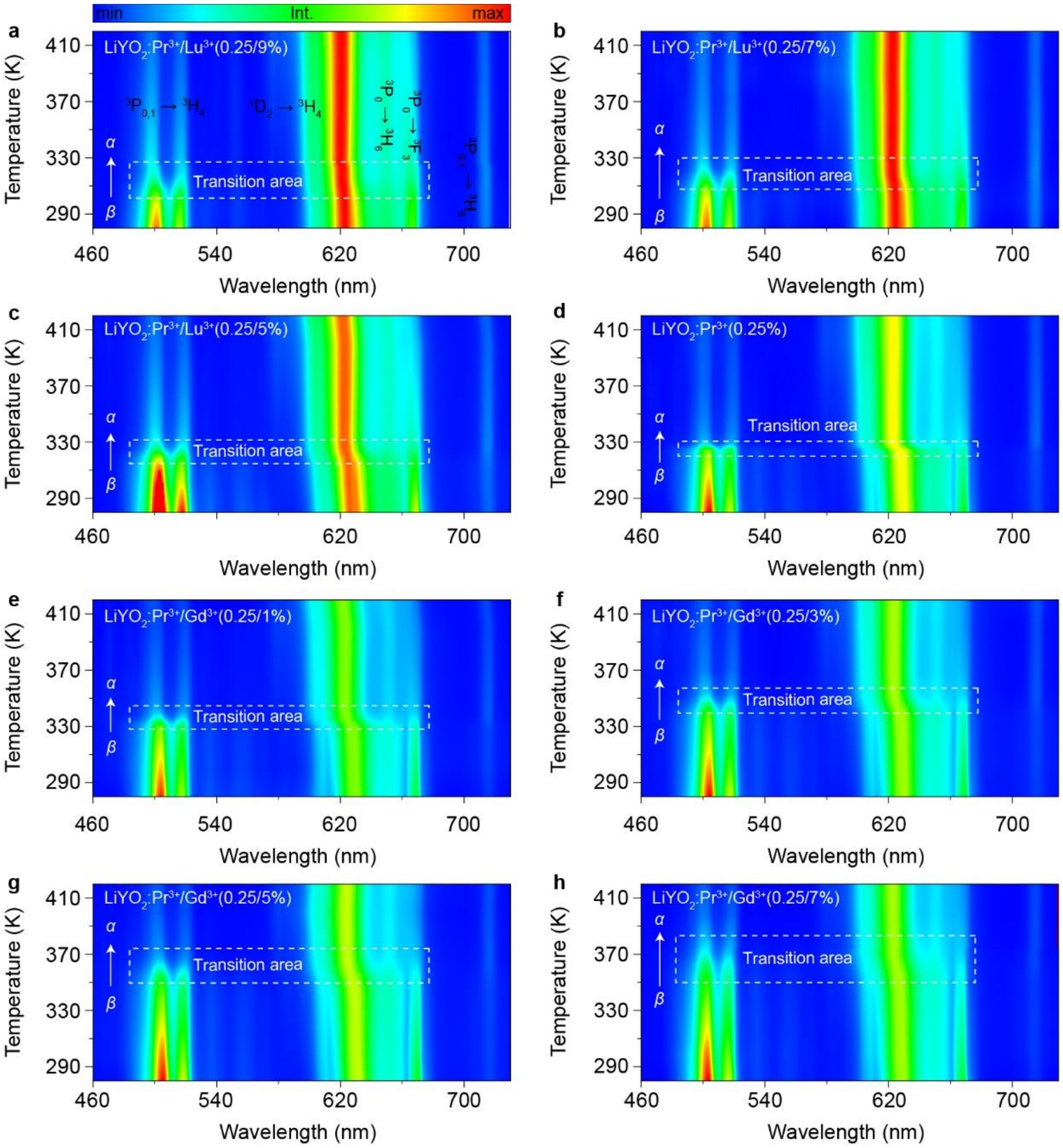

Figure S14. Normalized PL spectral mapping of **a)** $LiYO_2$:Pr^{3+}/Lu^{3+} (0.25/9%), **b)** $LiYO_2$:Pr^{3+}/Lu^{3+} (0.25/7%), **c)** $LiYO_2$:Pr^{3+}/Lu^{3+} (0.25/5%), **d)** $LiYO_2$:Pr^{3+} (0.25%), **e)** $LiYO_2$:Pr^{3+}/Gd^{3+} (0.25/1%), **f)** $LiYO_2$:Pr^{3+}/Gd^{3+} (0.25/3%), **g)** $LiYO_2$:Pr^{3+}/Gd^{3+} (0.25/5%), and **h)** $LiYO_2$:Pr^{3+}/Gd^{3+} (0.25/7%) as a function of temperatures (280-420 K) under 280 nm excitation.

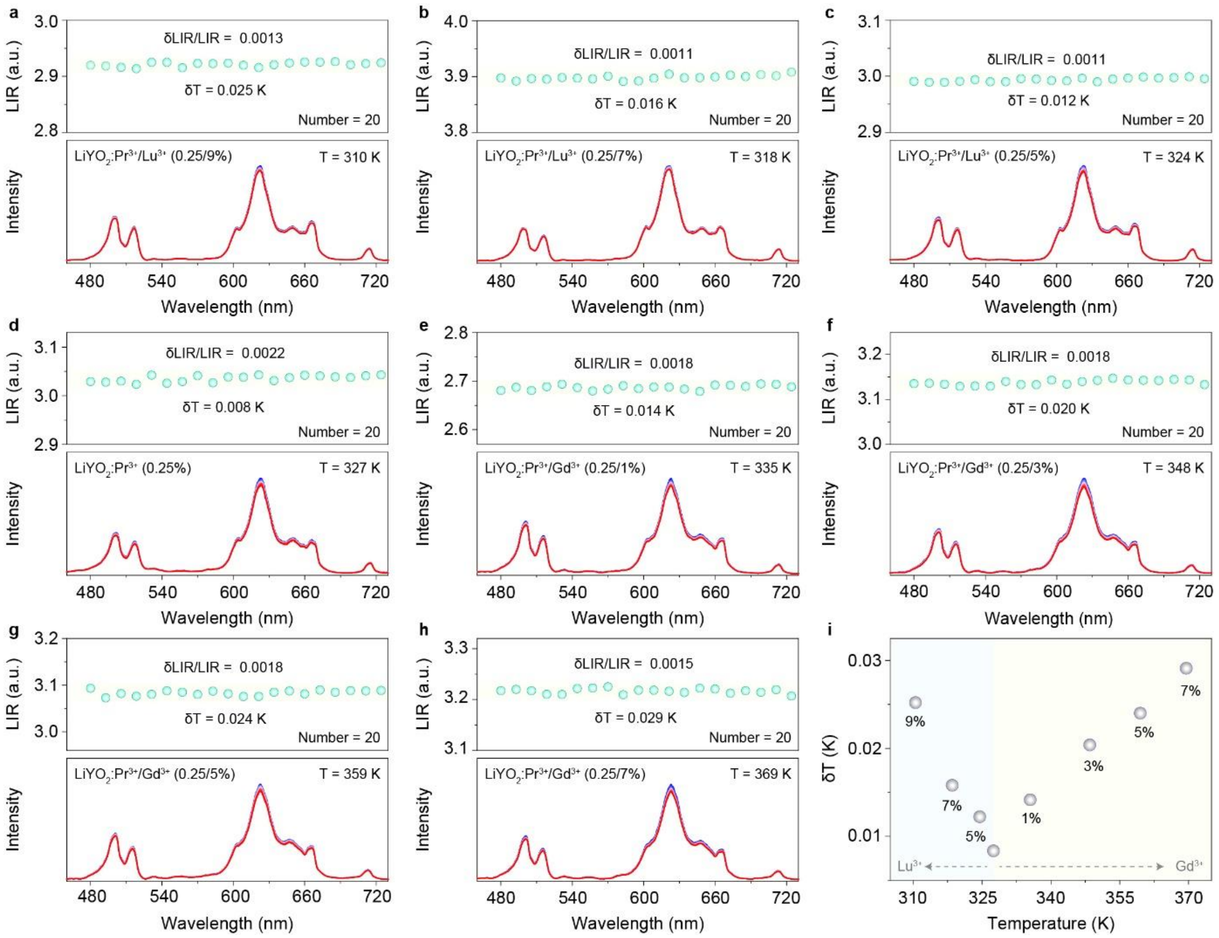

Figure S15. PL spectra measured for 20 times under 280 excitation (bottom) and fluctuation of LIR values (top) of **a)** $LiYO_2:Pr^{3+}/Lu^{3+}$ (0.25/9%) at 310 K, **b)** $LiYO_2:Pr^{3+}/Lu^{3+}$ (0.25/7%) at 318 K, **c)** $LiYO_2:Pr^{3+}/Lu^{3+}$ (0.25/5%) at 324 K, **d)** $LiYO_2:Pr^{3+}$ (0.25%) at 327 K, **e)** $LiYO_2:Pr^{3+}/Gd^{3+}$ (0.25/1%) at 335 K, **f)** $LiYO_2:Pr^{3+}/Gd^{3+}$ (0.25/3%) at 348 K, **g)** $LiYO_2:Pr^{3+}/Gd^{3+}$ (0.25/5%) at 359 K, and **h)** $LiYO_2:Pr^{3+}/Gd^{3+}$ (0.25/7%) at 369 K. **i)** The calculated optimal δT values of the samples as a function of temperature. In specific, the optimal δT values were found to be less than 0.03 K over a wide temperature range (310-370 K). The detailed thermometric performances of all samples are listed in **Table S5**.

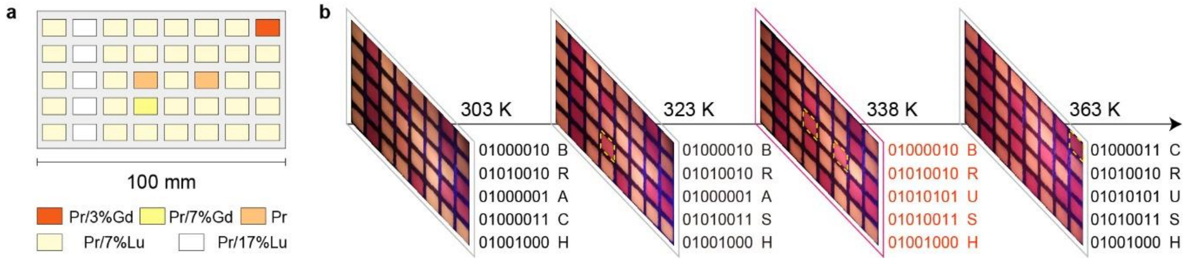

Figure S16. a) Schematic illustration of the pixelated patterns composed of $LiYO_2:Pr^{3+}$ (0.25%), $LiYO_2:Pr^{3+}/Lu^{3+}$ (0.25/17% and 0.25/7%), $LiYO_2:Pr^{3+}/Gd^{3+}$ (0.25/3% and 0.25/7%). We made a pixelated pattern (5*8 dot matrix) using different $LiYO_2$ crystals. With the yellow and red emissions as binary codes of "0" and "1", the pattern was designed to store ASCII characters that can be protected with temperature. **b)** Photographs of the pixelated patterns under the excitation of a handheld 275 nm lamp at 303, 323, 338, and 363 K, respectively. The dashed box indicates the region where PL switching occurs. The cryptographic information of "BRUSH" was only displayed at 338 K, where all samples were switched to the prescribed phases and thus the right emission colors. By contrast, false information of "BRACH", "BRASH", and "CRUSH" was decoded at 303, 323, and 363 K, due to undesired phase-transition in the samples.

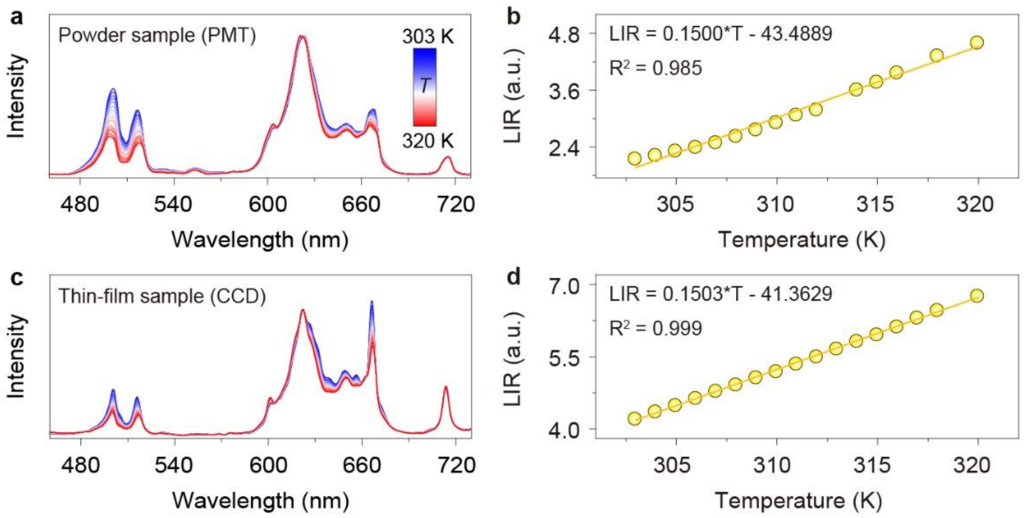

Figure S17. Normalized PL spectra and the calculated LIR values of **a-b)** powder sample and **c-d)** thin-film sample of $LiYO_2:Pr^{3+}/Lu^{3+}$ (0.25/9%) at different temperatures in the 303–320 K range. Note that the PL spectra of powder and thin-film samples were recorded by a visible photomultiplier tube (PMT) detector (200-900 nm) and a CCD (200-1100 nm), respectively. It can be found that LIR values can be linearly fitted against the temperature in both powder and thin-film samples with a similar slope. It is worth noting that the slight differences in peak shape and LIR value at a given temperature may stem from the different spectral responses between PMT and CCD detectors. As a result, there was a slight difference in relative sensitivity between these two detectors. For example, the calculated S_r values at 310 K were around 5.3% and 2.9% K^{-1} according to the equation S1 for PMT and CCD detectors, respectively.

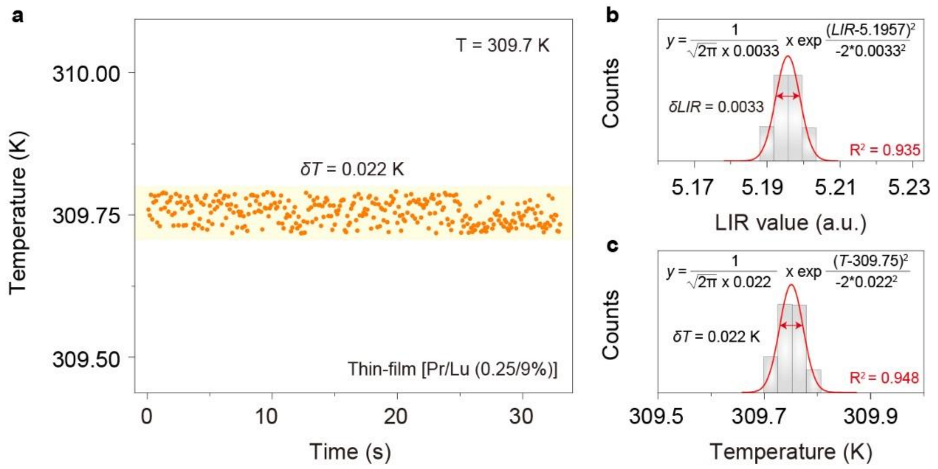

Figure S18. a) Fluctuation of temperature values derived from PL spectra of thin-film sensors at a constant temperature of 309.7 K during the continuous collection of PL spectra (acquisition time: 0.1 s). **b)** Histograms of the measured LIR values and **c)** the corresponding temperatures derived from the calibration curve. Note that these histograms were fitted using the probability density function of normal distribution:

$$y = \frac{1}{\sqrt{2\pi}\sigma} e^{-(x-\mu)^2/2\sigma^2} \quad \text{(Eq S3)}$$

where σ and μ denote standard deviation and average value, respectively. By converting LIR values into temperature using the measured calibration curve (LIR = 0.1503*T – 41.3629), the δT value was calculated to be around 0.022 K from the histogram of temperatures. This value was identical to the δT value (0.022 K) derived from the $\delta LIR/LIR$ value (~ 0.00064) and S_r value (~ 2.9% K^{-1}) at 309.7 K according to equation S2. Note that the deviation from a Gaussian distribution in the temperature fluctuations might stem from the subtle temperature variation of the human body during the spectral measurements.

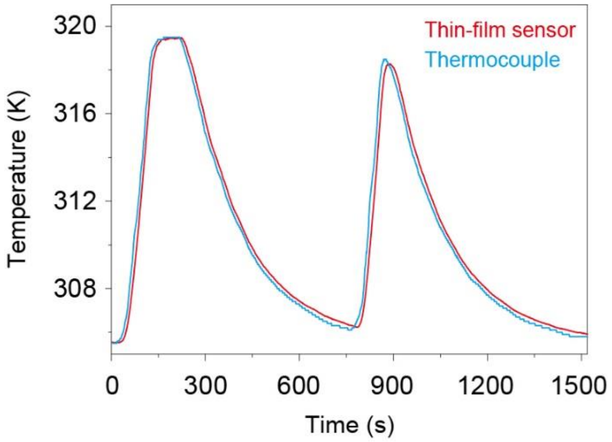

Figure S19. Real-time temperature detection using thin-film thermometer and thermocouple during the heating and cooling process. The as-prepared thin film can be uniformly heated and cooled by an external heating plate equipped with a water cooling system. Meanwhile, a K-type thermocouple probe was used to measure the temperature changes of the thin film as the control data. Notably, the thin-film sensor offered similar temperature results and response time in comparison with a commercial thermocouple.

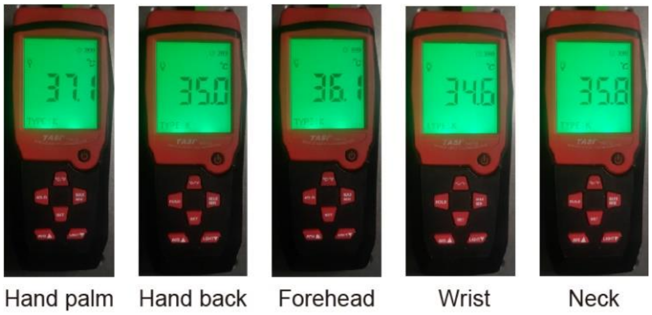

Figure S20. Measured temperatures of the human body parts by a K-type thermocouple fitting on the hand palm, back of hand, forehead, wrist, and neck. All temperature data of the human body were measured continuously for 10 s.

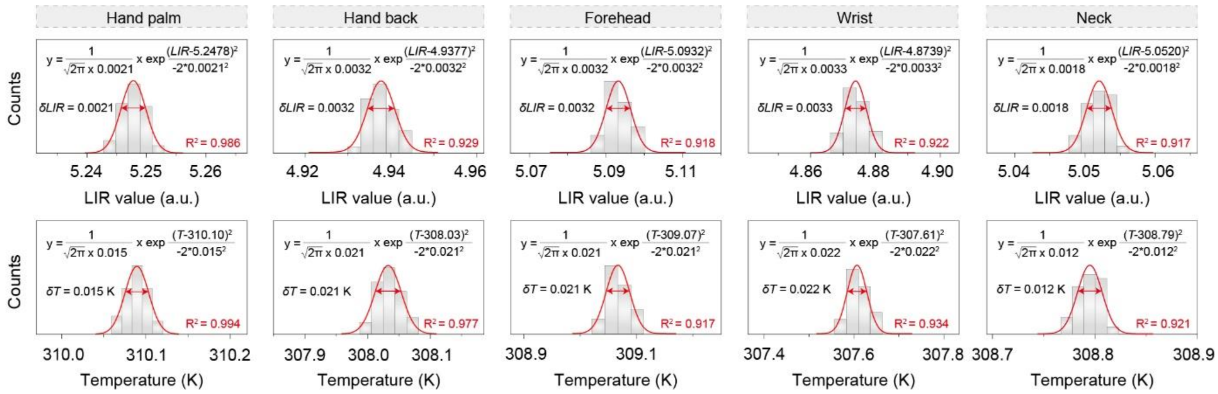

Figure S21. Histograms of the measured LIR values and the corresponding temperatures derived from the calibration curve (LIR = 0.1503*T – 41.3629) for different human body parts (hand palm, back of hand, forehead, wrist, and neck).

Table S1. Rietveld refinement and the calculated crystallographic results for Pr^{3+} doped β-$LiYO_2$ and α-$LiYO_2$ crystal.

Phase	β	α
**Space group**	$P2_1/c$	$I4_1/amd$
Z	4	4
a (Å)	6.12691	4.4379
b (Å)	6.19006	4.4379
c (Å)	6.20863	10.36187
V (Å^3)	206.386	204.076
R_{wp}	7.61%	4.73%
R_p	5.91%	3.22%

Table S2. The calculated formation energy (E_{form}) in β-$LiYO_2$ crystal upon Sc^{3+}, Lu^{3+}, Yb^{3+}, Dy^{3+}, Tb^{3+}, Gd^{3+} doping.

**Crystal phase**	**Dopant ions**	**E_{T2} (eV)**	**E_{T1} (eV)**	**E_{Y} (eV)**	**E_{dopant} (eV)**	**E_{form} (eV)**
β-$LiYO_2$	Sc → Y (CN=6)	-239.216	-239.381	-6.475	-6.475	0.03
	Lu → Y (CN=6)	-237.562			-6.475	0.01
	Yb → Y (CN=6)	-231.311			-6.475	3.21
	Dy → Y (CN=6)	-237.632			-6.475	-0.12
	Tb → Y (CN=6)	-237.580			-6.475	-0.04
	Gd → Y (CN=6)	-246.782			-6.475	-9.32

Table S3. Rietveld refinement and the calculated crystallographic results for β-$LiYO_2:Pr^{3+}$ co-doped with different concentrations of Lu^{3+} ions.

Lu^{3+} content	5%	7%	9%	17%
**Space group**		*(P2₁/c, I4₁/amd)*		*I4₁/amd*
Z		4		
***a* (Å)**	(6.1160, 4.4322)	(6.1077, 4.4262)	(6.1076, 4.4241)	4.4104
***b* (Å)**	(6.1881, 4.4322)	(6.1896, 4.4262)	(6.1528, 4.4241)	4.4104
***c* (Å)**	(6.19676, 10.3267)	(6.1786, 10.3056)	(6.1661, 10.3083)	10.2426
***V* (Å³)**	(205.653, 202.858)	(204.942, 201.895)	(203.378, 201.764)	199.231
R_{wp}	8.32%	9.94%	18.39%	8.11%
R_p	6.24%	7.57%	11.92%	6.17%
β/α	0.7684	0.6849	0.327	0

Table S4. Rietveld refinement and the calculated crystallographic results for β-$LiYO_2:Pr^{3+}$ co-doped with different concentrations of Gd^{3+} ions.

**Gd^{3+} content**	**1%**	**3%**	**5%**	**7%**
**Space group**		$P2_1/c$		
Z		4		
***a* (Å)**	6.13196	6.13492	6.14012	6.14043
***b* (Å)**	6.19144	6.1889	6.18715	6.18985
***c* (Å)**	6.21565	6.22002	6.22727	6.22637
***V* (Å^3)**	206.796	206.902	207.182	207.239
**R_{wp}**	11.98%	5.79%	5.34%	6.92%
**R_p**	9.16%	4.47%	4.15%	5.15%
β/α	1	1	1	1

Table S5. Phase transition, PL switching, and thermometric performance of the samples.

**sample**	**T-DSC (K)**	**T-PL (K)**	**S_{r-max} (K^{-1})**	**δT_{max} (K)**
$LiYO_2:Pr^{3+}/Lu^{3+}$ (0.25/9%)	285-320 K (294 K)	298-318 K (310 K)	5.3% (310 K)	0.025
$LiYO_2:Pr^{3+}/Lu^{3+}$ (0.25/7%)	301-327 K (309 K)	304-323 K (318 K)	6.9% (318 K)	0.016
$LiYO_2:Pr^{3+}/Lu^{3+}$ (0.25/5%)	306-330 K (315 K)	315-329 K (324 K)	9.2% (324 K)	0.012
$LiYO_2:Pr^{3+}$ (0.25%)	320-340 K (325 K)	322-333 K (327 K)	26.1% (327 K)	0.008
$LiYO_2:Pr^{3+}/Gd^{3+}$ (0.25/1%)	327-348 K (332 K)	329-343 K (335 K)	12.7% (335 K)	0.014
$LiYO_2:Pr^{3+}/Gd^{3+}$ (0.25/3%)	336-355 K (343 K)	340-355 K (348 K)	8.9% (348 K)	0.020
$LiYO_2:Pr^{3+}/Gd^{3+}$ (0.25/5%)	346-373 K (353 K)	350-373 K (359 K)	7.5% (359 K)	0.024
$LiYO_2:Pr^{3+}/Gd^{3+}$ (0.25/7%)	342-375 K (359 K)	355-386 K (369 K)	5.3% (369 K)	0.029

Table S6. Comparison of thermometric performance among the reported luminescent thermometers

Materials	Emissions (nm)	Working range (K)	S_r (K^{-1})	$S_{r\text{-}max}$ (K^{-1})	δT_{max} (K)	Ref
**$NaLuF_4$:Yb, Er**	Er^{3+}: 525/545	275-348	/	1% (303 K)	0.5	S3
**$(NaGdF_4:Er@NaYF_4)_n$**	Er^{3+}: 525/545	298-348	> 2%	3.02% (298 K)	/	S4
**$BaTiO_3$ Ho, Yb**	Ho^{3+}: 490/550	298-578	> 0.1%	2.78% (429 K)	0.85	S5
**$LaPO_4$: Nd^{3+}/Yb^{3+}**	Nd^{3+}: 750/865	280-490	> 1.14%	3.51% (280 K)	0.02	S6
**$LiErF_4$@$LiYF_4$**	Er^{3+}: 668/550	298-573	> 1.5%	5.27% (298 K)	/	S7
**Sr_2GeO_4:Pr**	Pr^{3+}: 295/630	17-600	> 0.30%	9.0% (22 K)	0.1	S8
**$NaYF_4$:Yb, Nd @$NaYF_4$@$NaYF_4$:Yb, Er**	Nd^{3+}: 803/Er^{3+}:654	300-435	> 2.3%	9.6% (300 K)	1	S9
**$NaYF_4$:Yb, Er/ $Yb_2W_3O_{12}$:Tm**	Tm^{3+}: 796/Er^{3+}:540	313-553	/	23.84% (380 K)	0.0042	S10
**Li_2ZnSiO_4:Mn**	Mn^{2+}: 530/650	253-373	> 0.01%	0.848% (373 K)	0.2	S11
**$Ca_6Ba(PO_4)_4O$:Mn**	Mn^{5+}: 1020/1140	303-350	> 1.3%	2.35% (303 K)	0.2	S12
**$Li_{1.97}Zn_{1.0292}G_{e3}O_8$: Cr**	Cr^{3+}: 713/721	50-300	> 0.36%	13.09% (50 K)	0.03	S13
**Ag/Ag_2S**	1311/1235	288-323	/	2% (323 K)	0.2	S14
**TTA-Nd-NPs**	TTA:550/Nd^{3+}:1075	283-323	>0.2%	7.1% (298 K)	0.1	S15
**Cdots&RB@ZIF-8^2-MMM**	Cdot:490/RB:580	293-353	> 0.52%	0.74% (293 K)	0.04	S16
**Ln-MOF**	Tb^{3+}:545/Eu^{3+}:617	90-240	> 0.6%	3.74% (240 K)	0.69	S17
**RCDs**	RCDs: 650	277-353	> 0.5%	1.2% (277 K)	0.5	S18
**$NaGdF_4$:Er, Yb@$NaGdF_4$**	Er^{3+}: τ_{540}	293-316	>1.9%	2.4% (293 K)	0.35	S19
**PbS QDs/Tm UCNPs**	Tm^{3+}&PbS: τ_{810}	293-323	>2.1%	5.6% (293 K)	0.5	S20
**$Sc_2(MoO_4)_3$:Yb/Er**	Er^{3+}: τ_{1550}	298-623	> 0.74%	12.33% (298 K)	0.11	S21
**TICT@AIE**	$\tau_{527\text{-}591}$	298-315	> 1.02%	17.72% (310 K)	0.04	S22
**$LiYO_2$:Pr^{3+}/Lu^{3+} or Gd^{3+}**	**Pr^{3+}: 503/630**	**310-370**	**> 5%**	**26.1% (327 K)**	**0.008**	**This work**

Reference

[1] J. J. Zhou, B. D. Rosal, D. Jaque, S. Uchiyama, D. Y. Jin**,** *Nat. Methods*. **2020**, *17*, 967.

[2] T. P. van Swieten, A. Meijerink, F. T. Rabouw, *ACS Photonics* **2022**, *9*, 1366.

[3] X. J. Zhu, W. Feng, J. Chang, Y. W. Tan, J. C. Li, M. Chen, Y. Sun, F. Y. Li, *Nat. Commun.* **2016**, *7*, 10437.

[4] X. F. Wu, S. P. Zhan, J. B. Han, Y. X. Liu, *Nano Lett.* **2021**, *21*, 272.

[5] T. Zheng, M. Runowski, I. R. Martín, S. Lis, M. Vega, J. Llanos, *Adv. Opt. Mater.* **2021**, *9*, 2100386.

[6] H. Suo, X. Q. Zhao, Z. Y. Zhang, C. F. Guo, *Chem. Eng. J*. **2020**, *389*,124506.

[7] L. Y, J. S. Huang, Z. C. An, Q. Y. Zhang, B. Zhou, *Nano Lett*. **2022**, *22*, 7042.

[8] C. D. S. Brites, K. Fiaczy, J. F. C. B. Ramalho, M. Sójka, L. D. Carlos, E. Zych, *Adv. Opt. Mater.* **2018**, *6*, 1701318.

[9] C. Mi, J. J. Zhou, F. Wang, G. G. Lin, D. Y. Jin, *Chem. Mater.* **2019**, *31*, 9480.

[10] Y. B. Wang, L. Lei, R. G. Ye, G. H. Jia, Y. J. Hua, D. G. Deng, S. Q. Xu, *ACS Appl. Mater. Interfaces.* **2021**, *13*, 23951.

[11] E. H. Song, M. H. Chen, Z. T. Chen, Y. Y. Zhou, W. J. Zhou, H. T. Sun, X. F. Yang, J. L. Gan, S. Ye, Q. Y. Zhang, *Nat. Commun*. **2022**, *13*, 2166.

[12] M. D. Dramićanin, Ł. Marciniak, S. Kuzman, W. Piotrowski, Z. Ristić, J. Periša, I. Evans, J. Mitrić, V. Đorđević, N. Romčević, M. G. Brik, C. G. Ma, *Light Sci. Appl.* **2022**, *11*, 279.

[13] R. Li, G. H. Wei, Z. J. Wang, Y. Wang, J. H. Li, S. X. He, L. P. Li, H. Suo, W. G. Ding, P. L. Li, *Laser Photonics Rev.* **2023**, *17*, 2200589.

[14] D. Ruiz, B. D. Rosal, M. Acebrón, C. Palencia, C. Sun, J. Cabanillas-González, M. López-Haro, A. B. Hungría, D. Jaque, B. H. Juarez, *Adv. Funct. Mater.* **2017**, *27*, 1604629.

[15] M. Xu, X. M. Zou, Q. Q. Su, W. Yuan, C. Cao, Q. H. Wang, X. J. Zhu, W. Feng, F. Y. Li, *Nat. Commun.* **2018**, *9*, 2698.

[16] Y. Y. Ding, Y. T. Lu, K. L. Yu, S. Wang, D. Zhao, B. L. Chen, *Adv. Opt. Mater.* **2021**, *9*, 2100945.

[17] T. T. Feng, Y. X. Ye, X. Liu, H. Cui, Z. Q. Li, Y. Zhang, B. Liang, H. R. Li, B. L. Chen, *Angew. Chem. Int. Ed.* **2020**, *59*, 21752.

[18] Y. L. Xu, Y. Yang, S. Lin, L. H. Xiao, *Anal. Chem.* **2020**, *92*, 15632.

[19] X. L. Liu, A. Skripka, Y. M. Lai, C. Jiang, J. D. Liu, F. Vetrone, J. Y. Liang, *Nat. Commun.* **2021**, *12*, 6401.

[20] X. C. Qiu, Q. W. Zhou, X. J. Zhu, Z. G. Wu, W. Feng, F. Y. Li, *Nat. Commun.* **2020,** *11*, 4.

[21] J. S. Liao, M. H. Wang, F. L. Lin, Z. Han, B. Fu, D. Tu, X. Y. Chen, B. Qiu, H. R. Wen, *Nat. Commun.* **2022**, *13*, 2090.

[22] K. Xue, C. Wang, J. X. Wang, S. Y. Lv, B. Y. Hao, C. L. Zhu, B. Z. Tang, *J. Am. Chem. Soc.* **2021**, *143*, 14147.
